# Transcriptional Profiling of Growth Hormone–Secreting Pituitary Adenoma Stem‐Like Cells: Unveiling the Significant Role of CXCR4 in Tumorigenesis and Clinical Applications

**DOI:** 10.1002/mco2.70946

**Published:** 2026-08-24

**Authors:** LinHao Yuan, Hao Yang, BaoWang Li, Huimin Sui, Peiliang Li, Xu Wang, Jiang Li, Guijun Jia, Zhaohui Zhu, Deling Li, Wang Jia, Peng Kang

**Affiliations:** ^1^ Department of Neurosurgery Beijing Tiantan Hospital Capital Medical University Beijing China; ^2^ Department of Nuclear Medicine State Key Laboratory of Complex Severe and Rare Diseases Beijing Key Laboratory of Molecular Targeted Diagnosis and Therapy in Nuclear Medicine Peking Union Medical College Hospital, Chinese Academy of Medical Sciences Peking Union Medical College Beijing China; ^3^ Department of Neurosurgery Beijing Ditan Hospital Capital Medical University Beijing China; ^4^ China National Clinical Research Center for Neurological Diseases Beijing China

**Keywords:** AMD3465, CXCR4, ^68^Ga‐Pentixafor, growth hormone–secreting pituitary adenoma, human pituitary adenoma stem‐like cells, RNA sequencing

## Abstract

Growth hormone–secreting pituitary adenoma (GHPA) can cause excessive growth hormone production, which can lead to clinical symptoms such as acromegaly. Tumor stem cells (TSCs) are postulated to play a significant role in the development of GHPA. We performed RNA sequencing on human pituitary adenoma stem‐like cells (hPASCs) and matched bulk tumors to map a differentiation protein–protein interaction (PPI) network. The function of the C‐X‐C motif chemokine receptor‐4 (CXCR4) gene was tested in hPASCs and the GH3 cell line. The CXCR4‐based ^68^Ga‐pentixafor tracer was assessed by the positron emission tomography and computed tomography (PET/CT) scanning of GHPA patients. A total of 685 differentially expressed genes were identified between hPASCs and differentiated tumor cells. Four clusters of genes were predicted, each carrying distinct biological functions. *CXCR4* was pinpointed as a potential hub gene, which significantly affected the self‐renewal and differentiation of hPASCs, as well as the proliferation, invasion, and migration of GH3 cells. *CXCR4* suppression decreased intra‐tumoral angiogenesis and epithelial‐to‐mesenchymal transition (EMT). CXCR4‐based ^68^Ga‐pentixafor was a more adaptive tracer than ^18^F‐FDG in diagnosing GHPA using PET/CT. This study provides a comprehensive genetic profiling of hPASCs and substantiates the critical role of *CXCR4* in tumorigenesis, highlighting its potential in translational medicine.

## Graphical abstract

Growth hormone–secreting pituitary adenomas harbor hPASCs (human pituitary adenoma stem‐like cells). Genetic profiling of hPASCs identified CXCR4 as a significant gene orchestrating a regulatory network in the self‐renewal and differentiation of hPASCs, as well as the modulation of tumor cells and the microenvironment. CXCR4‐based radiotracer ^68^Ga‐pentixafor can be an adaptive imaging agent for PET/CT assessment in clinical practice.

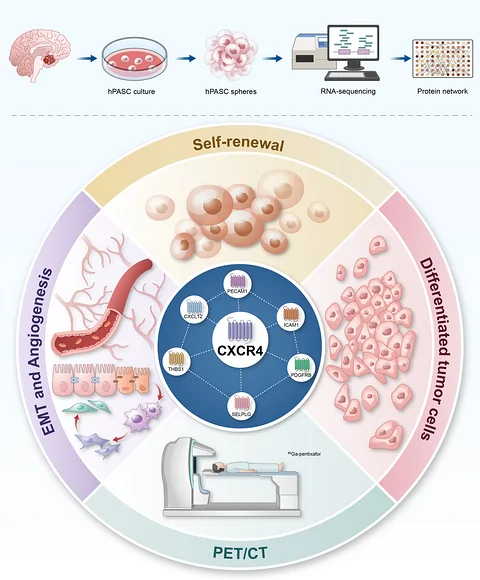

## 1 Introduction

Growth hormone–secreting pituitary adenoma (GHPA), or growth hormone–secreting pituitary neuroendocrine tumor (GH‐PitNET), represents a subtype of pituitary adenoma and comprises approximately 12% of the clinical cases [1, 2]. These tumors secrete excessive amounts of growth hormone (GH) into the bloodstream, elevating insulin‐like growth factor‐1 (IGF‐1) levels and triggering significant clinical symptoms such as gigantism and acromegaly. Mutations of guanine nucleotide binding protein, alpha stimulating (*GNAS*), the dysregulation of pituitary‐specific positive transcription factor 1 (PIT‐1), and the activation of phosphatidylinositol 3‐kinase/Ak strain transforming (PI3K/Akt) can play vital roles in GHPA pathogenesis [3]. Other factors, such as epigenetic abnormalities and microenvironmental factors, may influence these alterations, ultimately leading to tumor formation [3].

Tumor stem cells (TSCs) were first isolated and identified in leukemia using CD34^+^ and CD38^−^ sorting [4], and later were verified in many other types of tumors. Typically, they comprise a distinct cell subpopulation that exhibits stem cell‐like properties, such as self‐renewal and differentiation, and a certain degree of resistance to radiotherapy and chemotherapy, which contributes to tumor recurrence and metastasis [5]. In recent years, human pituitary adenoma stem‐like cells (hPASCs), a type of TSC, have been implicated in the tumorigenic mechanisms of GHPA [6, 7]. The existence of hPASCs was substantiated by the expression of general stem cell–associated genes, such as *SOX2*, *NESTIN*, and *CD133* [5], or by the side population technique [8]. For example, CD133+ cells in pituitary adenomas demonstrated self‐renewal and differentiation capacities [9]. Wurth revealed more details of the phenotypical characteristics of hPASCs in GHPA, including morphological changes, marker proteins, and differentiation [10]. Accumulated evidence has clarified certain hPASC characteristics; however, limited information regarding their genetic properties is available. Previously, we cultured hPASCs from gonadotrophic pituitary adenomas and described the genetic profiles between hPASCs and differentiated tumor cells [11].

C‐X‐C motif chemokine receptor‐4 (CXCR4) is recognized as a TSC marker and a pro‐tumoral gene in multiple human malignancies, including breast [12], colon [13], lung [14], and prostate cancers [15], as well as glioma [16]. CXCR4 has been reported to be differentially expressed among pituitary adenoma subtypes [17] and is significantly elevated in invasive adenoma compared with its noninvasive counterparts [18]. Furthermore, overexpression of *CXCR4* induced an increase in the proliferation of pituitary adenoma tumor cells, and this effect could be blocked by its antagonist, AMD3100 [19]. Although CXCR4 is present in both hPASCs and differentiated tumor cells [10], the molecular functions (MFs) and mechanisms by which *CXCR4* is involved in tumorigenesis remain unclear.

Based on prior evidence demonstrating the utility of the CXCR4‐targeting ^68^Ga‐pentixafor radiotracer in identifying a variety of tumors using clinical positron emission tomography (PET) imaging [20], one study has investigated it in ACTH‐secreting pituitary adenomas [21], while its use in other subtypes of pituitary adenomas remains undefined. As a result, a pilot study evaluating ^68^Ga‐pentixafor in GHPA patients is needed. The present study has two primary aims: (1) to examine the genetic profile of hPASCs and differentiated tumor cells, and (2) to identify the functional significance of *CXCR4* in hPASCs derived from GHPA and evaluate its clinical applications. We hope to verify the potential of *CXCR4* as both a diagnostic biomarker and a therapeutic target, thereby offering new avenues for treating GHPA.

## 2 Results

### 2.1 Culture, Verification, and Differentiation of hPASCs from Clinical GHPA Samples

**FIGURE 1:**
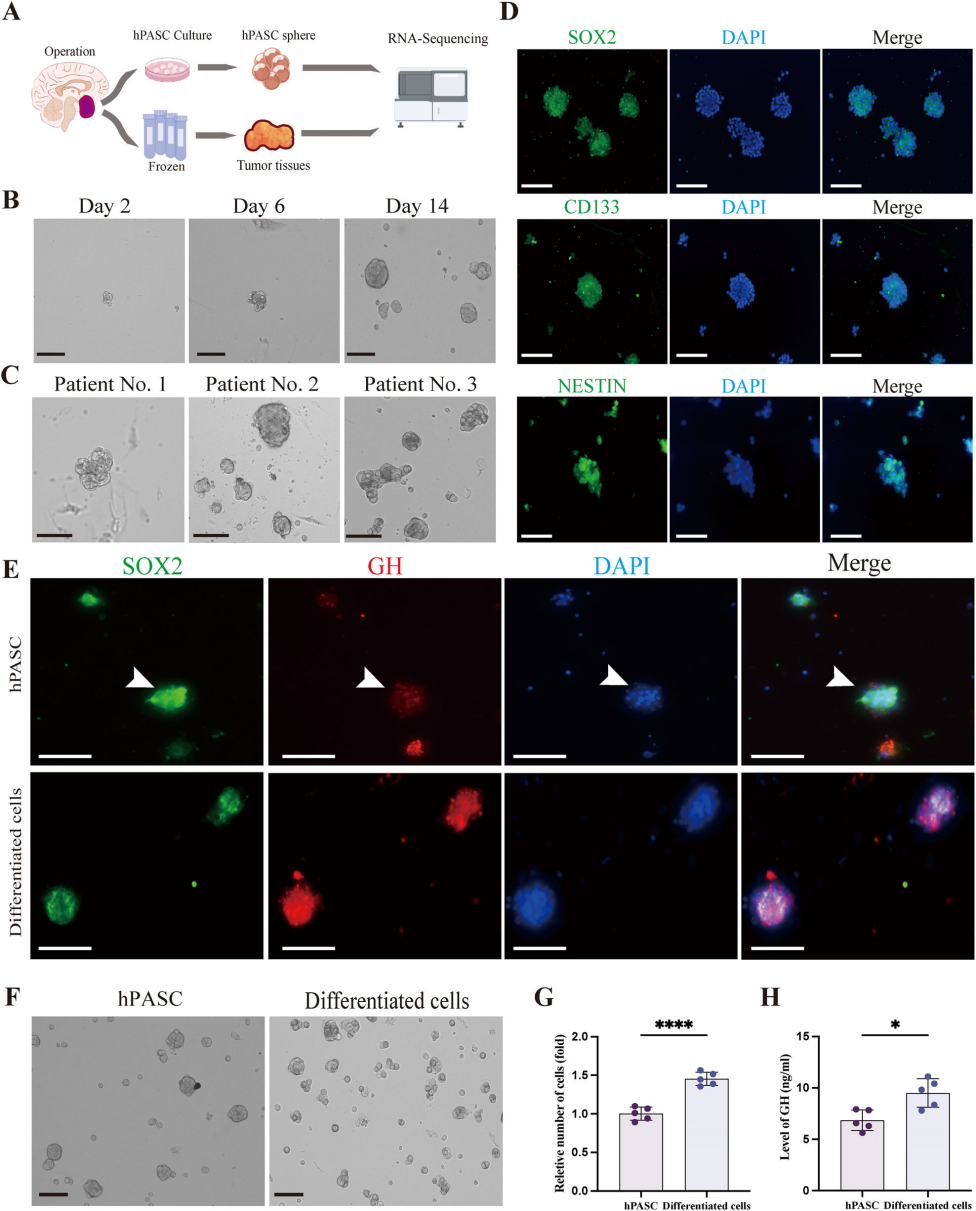
Morphology and verification of hPASCs from GHPA. (A) The diagram outlines the procedure of hPASC culture and cryopreservation for RNA sequencing. (B) Representative morphology of hPASCs from one clinical sample on Day 2, 6, and 14 by microscopy. (C) Morphology of hPASCs on Day 14 from three different clinical samples under microscopic observation. (D) Immunostaining of SOX2, CD133, and NESTIN in hPASCs. (E) Immunostaining of SOX2 and GH in hPASCs and the differentiated tumor cells. Differentiated cells by Fetal bovine serum (FBS) exhibited lower SOX2 levels but stronger GH immunoreactivity compared to the parental hPASCs. (F) Microscopic observation of hPASCs and the differentiated tumor cells. (G) Quantitation of cell number of hPASCs and the differentiated tumor cells. *N* = 5 independent experiments. (H) Quantitation of GH level in the culture medium of hPASCs and differentiated tumor cells by ELISA. *N* = 5 independent experiments. Scale bar is 100 µm for all panels. *p < 0.05, ****p < 0.001.

After a 14‐day culture in stem cell medium, single‐cell‐derived hPASCs formed spheroid aggregates (Figure 1B,C). Immunostaining confirmed the expression of generic stem cell markers SOX2, CD133, and NESTIN in hPASCs (Figure 1D). Exposure to Dulbecco's Modified Eagle Medium (DMEM)/F‐12 supplemented with 15% fetal bovine serum (FBS) for 5 days induced the differentiation of hPASCs. Immunohistochemical analysis showed relatively weak GH staining in hPASCs but markedly strong expression in differentiated cells (Figure 1E). The number of differentiated cells was increased by 45.26% relative to undifferentiated hPASCs (*p* < 0.001; Figure 1F–G), and GH secretion into the culture medium rose by 38.53% (*p* < 0.05; Figure 1H).

### 2.2 Screening of Differentially Expressed Genes (DEGs) in hPASCs and Enrichment Analysis

**FIGURE 2:**
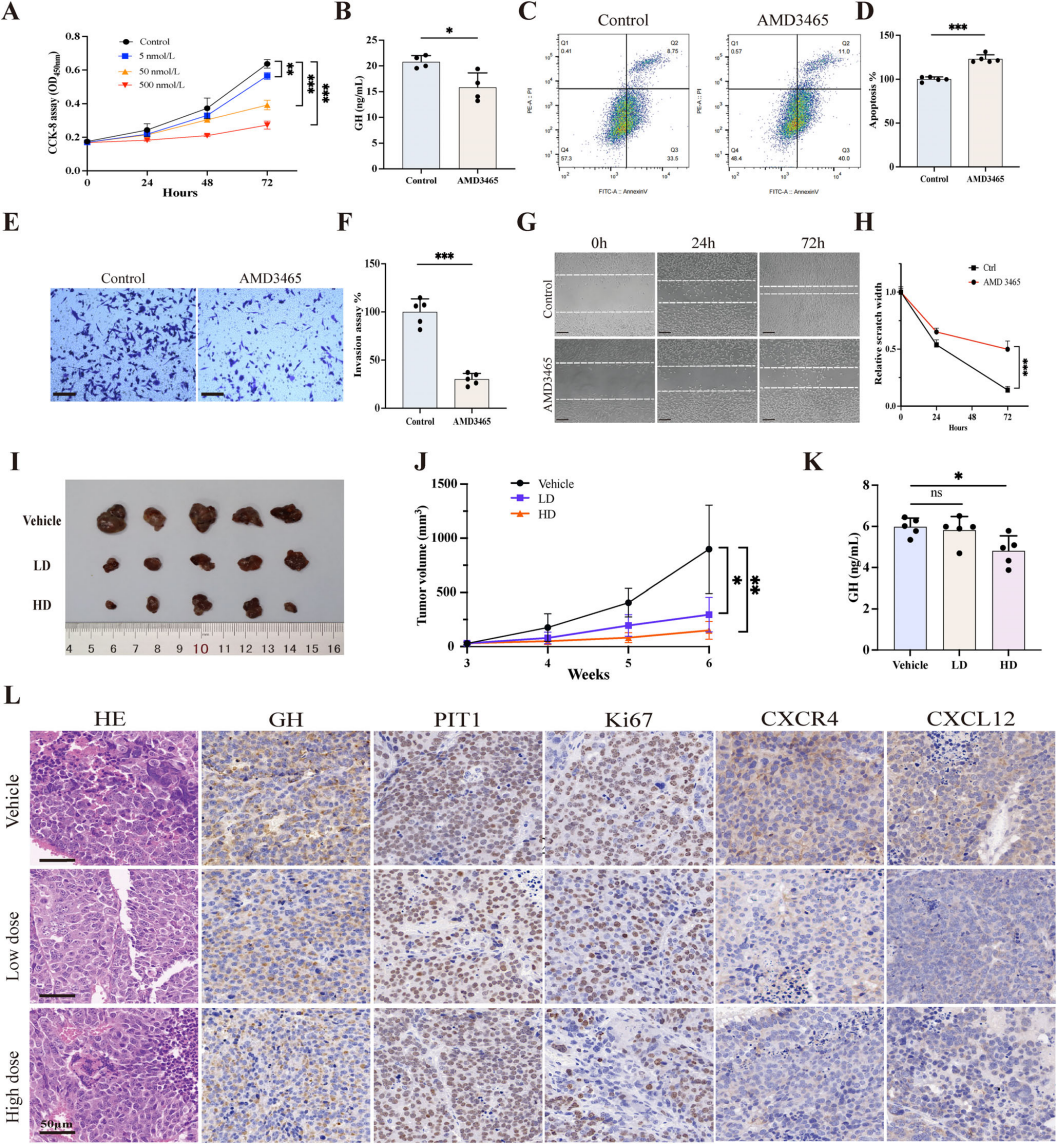
Differentially expressed genes (DEGs) and enrichment analysis of hPASCs and the matched bulky tumor cells. (A) PCA showed a distinctive expression pattern of hPASCs (blue) and tumor cells (red). (B) Volcano plot of DEGs. The upregulated genes (Log_2_FC > 0.5, *p*_adj_ < 0.05) were in red and the downregulated genes (Log_2_FC < −0.5, *p*_adj_ < 0.05) were in blue. (C) KEGG analysis of DEGs. GO analysis of DEGs, including (D) biological process, (E) molecular function, and (F) cellular component. (G) Top‐rated Cluster 1 contains seven putatively related genes. (H) KEGG analysis of DEGs in Cluster 1.

The genetic profiling of hPASCs and matched tumor cells was performed using RNA sequencing to identify DEGs. Principal component analysis showed distinctive separation between two groups based on global expression patterns (Figure 2A). In total, 685 DEGs met the thresholds (|log_2_FC| > 0.5; *p*_adj_ < 0.05), with 363 upregulated and 322 downregulated genes in the hPASC group (Figure 2B).

Kyoto Encyclopedia of Genes and Genomes (KEGG) pathway enrichment analysis (Figure 2C) highlighted transcriptional misregulation in cancer, Th17 cell differentiation, endocrine resistance, and steroid biosynthesis. Gene Ontology (GO) enrichment analysis (Figure 2D) identified the top biological processes (BPs), including the regulation of transcription, cell differentiation, apoptosis, cell proliferation, and migration, with additional enrichment for WNT signaling, vasculogenesis, stem cell population maintenance, PI3K, and dopamine secretion. MF indicated that protein kinase, hormone activity, and   Phosphatidylinositol‐3,4‐bisphosphate binding were involved. The implicated genes were predominantly enriched in the nucleus, cytoplasm, membranes, endoplasmic reticulum, Golgi apparatus, and lysosomes, as determined by subcellular localization (Figure 2F). Gene set enrichment analysis (GSEA) was also performed, with the results included in Figure S3.

A protein–protein interaction (PPI) network was constructed using the Search Tool for the Retrieval of Interacting Genes/Proteins (STRING) database to identify putative hub genes associated with hPASC differentiation, and it was visualized using Cytoscape (v3.7.2) (Figure S1). Network modules were detected using Molecular Complex Detection (MCODE), which identified cluster 1 as the top‐scoring candidate (Figure 2G). Cluster 1 comprised seven genes *CXCL12, CXCR4, PECAM1, ICAM1, PDGFRB, SELPLG*, and *THBS1*. KEGG enrichment of these genes implicated CXCL12‐mediated chemokine signaling and cell motility, extracellular exosome‐related processes, signaling receptor binding, and endothelial cell differentiation (Figure 2H). Three additional clusters and the enrichment results are provided in Figure S2.

As the ligand for CXCL12, *CXCR4* displayed high scores associated with other genes, as calculated using the MCODE algorithm (Table S1). *CXCR4* has been widely recognized as a pro‐tumoral gene and a TSC marker across multiple tumor types [22]; however, its role in hPASCs remains poorly understood. Therefore, we chose *CXCR4* for further examination.

### 2.3 Effects of the CXCR4 Antagonist AMD3465 on GH3 Cells

**FIGURE 3:**
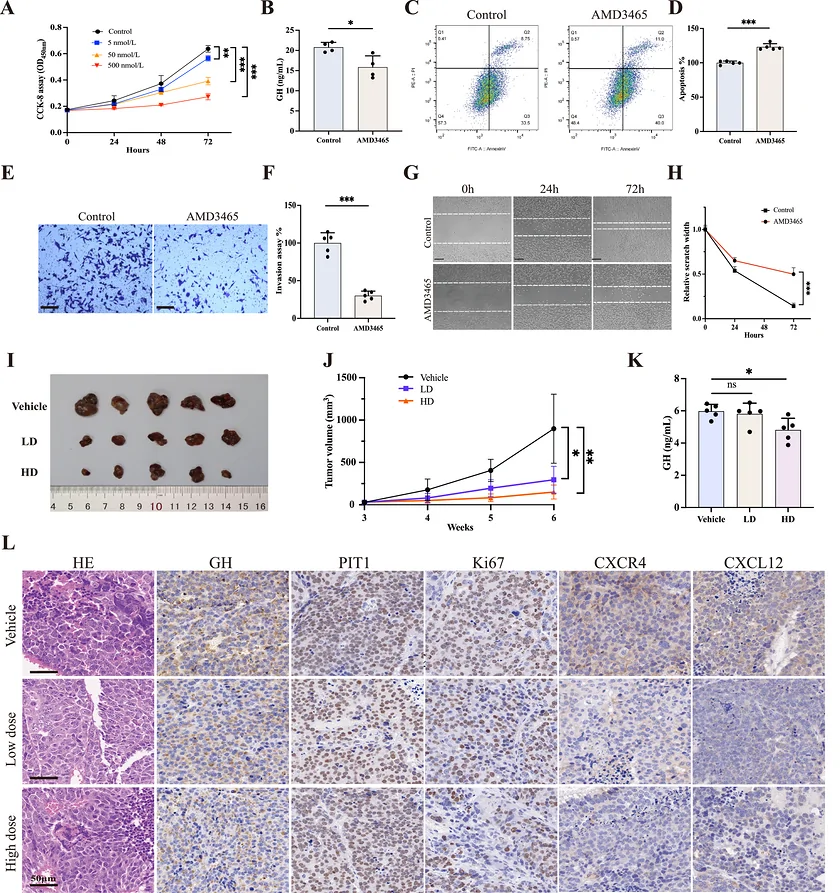
Effects of CXCR4 antagonist AMD3465 on GH3 cells. (A) Proliferation of GH3 cells upon treatment of AMD3465 at different concentrations. *N* = 3 independent experiments. (B) Quantitation of GH levels in the culture medium of GH3 cells in the presence of 50 nM AMD3465 by ELISA. *N* = 4 independent experiments. (C and D) Quantitation of apoptosis rate of GH3 cells exposed to 50 nM AMD3465 by flow cytometry. The apoptosis rate in the control group was set as 100%. *N* = 5 independent experiments. (E and F) Invasion assay of GH3 cells after treatment with AMD3465. *N* = 5 independent experiments. Scale bar is 50 um. (G and H) Migration assay of GH3 cells after treatment with AMD3465. The width of scratch on day 0 was set as 1. *N* = 3 independent experiments. (I) Representative image of gross tumors resected from GH3 xenograft mouse after treatment of AMD3465 at week 6. *N* = 5 animals. (J) Tumor volume of GH3 xenograft mice treated with AMD3465 from week 3 to week 6. *N* = 5 animals. (K) Measurement of GH levels in the blood serum of GH3 xenograft mice. *N* = 5 animals. (L) Immunostaining of GH, PIT1, Ki67, CXCR4, and CXCL12 in the tumor resected from the GH3 xenograft mice. LD: low dose (2 mg/kg); HD: high dose (4 mg/kg). *p < 0.05, **p < 0.01; ***p < 0.005.

We used AMD3465, a potent and highly selective CXCR4 antagonist [23], and first confirmed its effects on GH3 cells in vitro. After defining the AMD3465 dose–response curve (Figure S5), three concentrations (5, 50, and 500 nmol/L) were chosen, all of which significantly reduced GH3 cell proliferation compared with the control group (Figure 3A). After treatment of 50 nmol/L AMD3465, GH levels in the culture medium was decreased by 23.8% (*p* < 0.05; Figure 3B). In addition, AMD3465 increased the apoptosis rate by 23.1% (*p* < 0.005; Figure 3C,D), reduced the invasion of GH3 cells by 69.8% (*p* < 0.005; Figure 3E,F), and decreased the migration capacity by 35% (*p* < 0.005; Figure 3G,H).

Next, we established a GHPA xenograft model through the subcutaneous injection of GH3 cells into BALB/c nude mouse to evaluate the inhibitory effects of AMD3465 in vivo. The experimental procedure is illustrated in Figure S7A. Two doses (LD: low dose at 2 mg/kg; HD: high dose at 4 mg/kg) were used. AMD3465 significantly inhibited xenograft tumor volume by 67.10% in the LD group (*p* < 0.05) and 83.28% in the HD group (*p* < 0.01; Figure 3I,J), without affecting body weight (Figure S7B). GH levels were not changed in the LD group but were significantly reduced in the HD group (*p* < 0.05) (Figure 3K). Immunohistochemical analysis demonstrated a significant reduction in CXCR4 and CXCL12 staining in the xenograft tumor tissues in both groups (Figure 3L).

### 2.4 Effects of CXCR4 Antagonist AMD3465 on hPASCs

**FIGURE 4:**
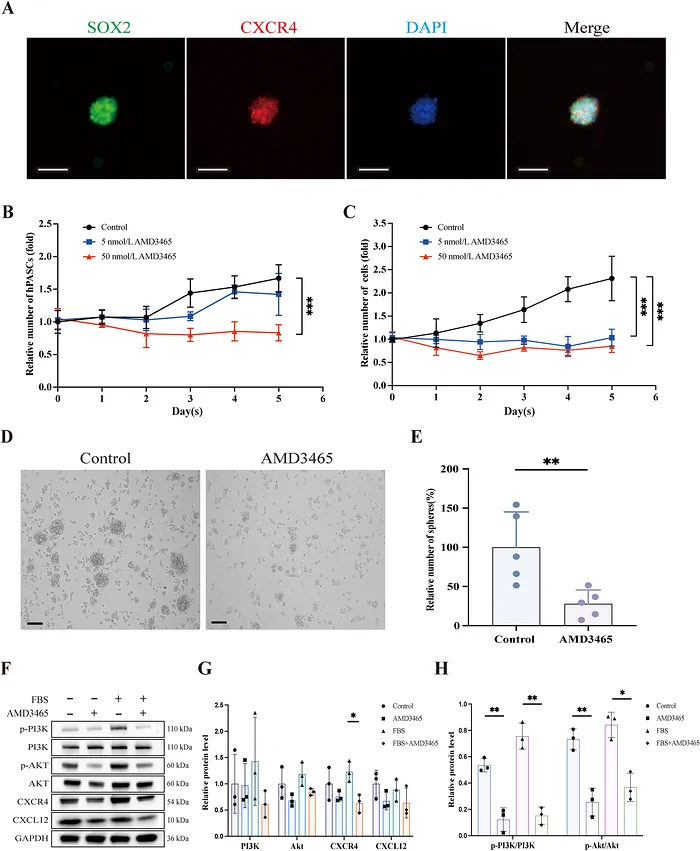
Effects of AMD3465 on hPASCs. (A) Co‐immunostaining of CXCR4 and SOX2 in hPASCs. Scale bar is 100 µm. (B) Cell proliferation of hPASCs in the presence of AMD3465. Cell number on Day 0 was set as 1. *N* = 4 independent experiments. (C) Cell number of the differentiated tumor cells from hPASCs in the presence of AMD3465 up to 5 days. Cell number on Day 0 was set as 1. *N* = 4 independent experiments. (D and E) Number of newly formed tumorspheres in the presence of 50 nM AMD3465 by sphere formation assay. The number of spheres in the control group was set as 100%. *N* = 5 independent experiments. Scale bar is 50 µm. (F) Representative images of p‐PI3K, PI3K, p‐Akt, Akt, CXCR4, and CXCL12 protein levels in hPASC and the differentiated tumor cells in vitro. p‐PI3K: phosphorylated‐PI3K; p‐Akt: phosphorylated‐Akt. (G) Quantitation of PI3K, Akt, CXCR4, and CXCL12 protein levels by densitometry. The density in untreated hPASC was set as 1. *N* = 3 independent experiments. (H) Quantitation of p‐PI3K/PI3K and p‐Akt/Akt. *N* = 3 independent experiments. *p < 0.05, **p < 0.01, *** *p* < 0.005.

Immunostaining showed that CXCR4 was positively co‐expressed with SOX2 protein in cultured hPASCs (Figure 4A). AMD3465 at 50 nmol/L significantly suppressed hPASC proliferation over 5 days of culture (Figure 4B). During differentiation, treatment with AMD3465 at both 5 and 50 nmol/L significantly reduced the number of newly generated differentiated tumor cells (Figure 4C). Sphere‐formation assays showed that 50 nmol/L AMD3465 significantly decreased the number of newly formed tumoroid spheres in vitro, indicating an impaired self‐renewal capacity of hPASCs (Figure 4D,E). Silencing of *CXCR4* in hPASCs had similar effects (Figure S6).

GO (Figure 2D) and GSEA analyses (Figure S3) showed that the PI3K/Akt signaling pathway was potentially involved in the differentiation of hPASCs, which is corroborated by previous reports [16, 24], indicating that the CXCL12/CXCR4 axis activates the PI3K signaling pathway. Considering this, we assessed the PI3K and Akt expression in hPASCs and their differentiated progeny driven by FBS. As shown in Figure 4F,G, AMD3465 significantly decreased phosphorylated‐PI3K (p‐PI3K) and phosphorylated‐Akt (p‐Akt) protein levels (Figure 4H), whereas it did not affect the total protein levels of PI3K or Akt (Figure 4G).

### 2.5 Effects of CXCR4 on the Tumor Microenvironment and Related Genes

**FIGURE 5:**
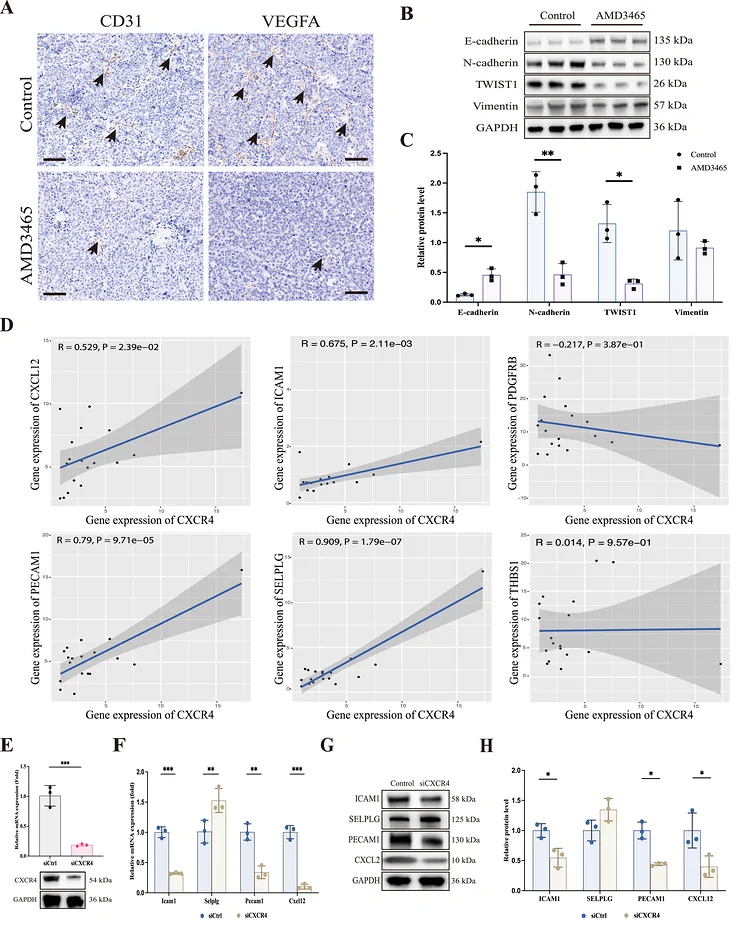
Effects of *CXCR4* on tumor microenvironment and its correlation with putative genes. (A) Immunostaining of CD31 and VEGFA in AMD3465‐treated GH3 tumors resected from xenograft mice. Scale bar is 50 µm. (B) Representative images of four EMT markers in hPASCs after treatment with AMD3465 by western blot. (C) Quantitation of E‐cadherin, N‐cadherin, TWIST1, and Vimentin protein levels. (D) Correlation analysis of mRNA levels of six putative with *CXCR4* from human GHPA clinical samples. *CXCL12*, *ICAM1*, *PECAM1*, and *SELPLG* were significantly correlated with *CXCR4*. N = 18 samples. (E) Confirmation of siRNA targeting *Cxcr4* (siCXCR4) at mRNA (upper panel) and protein level (lower panel) in GH3 cells. *N* = 3 independent experiments. (F) Quantitation of *Icam1*, *Selplg*, *Pecam1*, and *Cxcl12* mRNA levels after silencing *CXCR4* in GH3 cells. *N* = 3 independent experiments. (G) Representative images of ICAM1, SELPLG, PECAM1, and CXCL12 in the *Cxcr4‐*silenced GH3 cells. (H) Quantitation of ICAM1, SELPLG, PECAM1, and CXCL12 protein levels in the *Cxcr4‐*silenced GH3 cells. *N* = 3 independent experiments. *p < 0.05, **p < 0.01; ***p < 0.005.

Since pituitary adenoma is a complex entity, we next investigated if CXCR4 could affect some aspects of the tumor microenvironment, such as angiogenesis and epithelial‐mesenchymal transition (EMT). CD31+ and VEGFA+ immunostaining were significantly reduced in AMD3465‐treated GHPA xenograft tumor tissues generated from the GH3 mouse model (Figure 5A). In terms of EMT, AMD3465 significantly increased E‐cadherin expression and reduced N‐cadherin and TWIST1 levels in hPASCs (Figure 5B,C).

As shown in Figure 2G, *CXCR4* is putatively associated with six other genes in tumor formation, suggesting that *CXCR4* may exert its function through this relationship. We first tested if they were correlated with *CXCR4* in clinical GHPA samples. The quantitation of mRNA from human GHPA tissues showed that *CXCR4* was significantly associated with *CXCL12, ICAM1, PECAM1* (*CD31*), and *SELPLG* (*p* < 0.05; Figure 5D). After silencing *CXCR4* in GH3 cells using small interference RNA (siRNA) (Figure 5E), the mRNA levels of *Icam1*, *Pecam1*, and *Cxcl12* were significantly decreased, while *Selplg* expression was increased (Figure 5F). Quantification of protein levels verified the changes in ICAM1, PECAM1, and CXCL12, but not SELPLG (Figure 5G,H). In addition, cell proliferation and GH secretion were reduced in the *Cxcr4*‐silenced group (Figure S8).

### 2.6 Value of ^68^Ga‐Pentixafor for PET/CT Imaging in GHPA Patients

**FIGURE 6:**
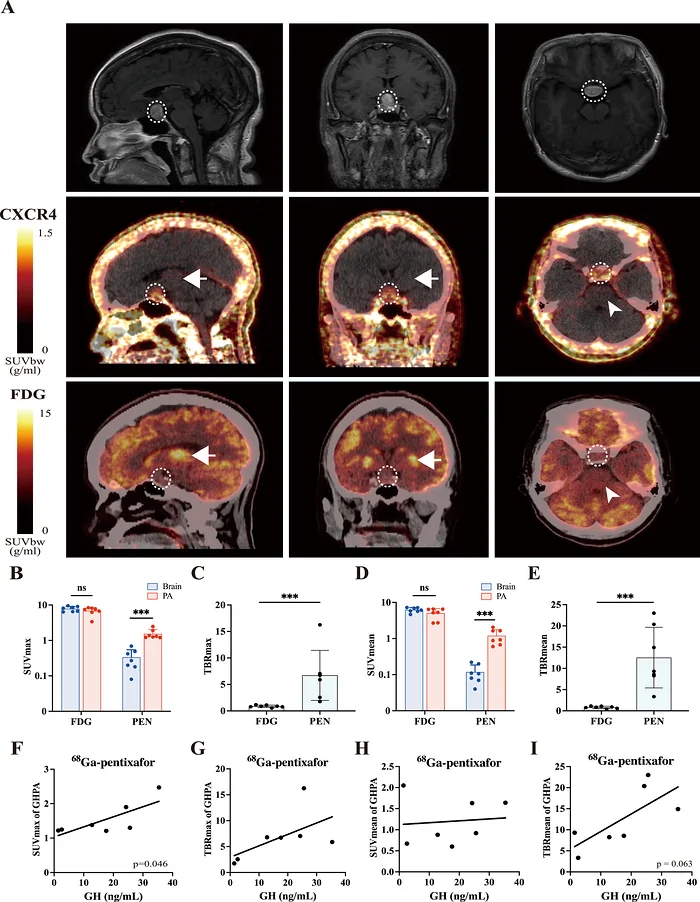
Evaluation of ^68^Ga‐pentixafor and ^18^F‐FDG in PET/CT in seven GHPA patients. (A) Enhanced MRI scans and preoperative ^68^Ga‐pentixafor and ^18^F‐FDG PET/CT images across coronal, axial, and sagittal planes. Arrows in the left and middle panels indicate the thalamus tissue. The arrowhead in the right panel points to the brainstem. (B–E) Quantitation of ^68^Ga‐pentixafor and ^18^F‐FDG in GHPA. (B) SUVmax, (C) TBRmax, (D) SUVmean, and (E) TBRmean. (F–I) Correlation of GH level with ^68^Ga‐pentixafor PET/CT quantitation. (F) Correlation of GH with SVUmax; (G) correlation of GH with TBRmax; (H) correlation of GH with SUVmean; and (I) correlation of GH with TBRmean. SUVmax: maximal standardized uptake value; SUVmean: mean of standardized uptake value; TBRmax: maximal tumor‐to‐brain ratio; TBRmean: mean of tumor‐to‐brain ratio. *p < 0.05, **p < 0.01, ***p < 0.005.

Given the expression pattern of CXCR4 in hPASCs and tumor cells, we investigated whether it could be translated into clinical usage. A pilot study was conducted enrolling seven GHPA patients (Table S4), utilizing the CXCR4‐targeted radiotracer ^68^Ga‐pentixafor compared to the conventional ^18^F‐fluorodeoxyglucose (^18^F‐FDG) tracer. As shown in Figure 6A, ^68^Ga‐pentixafor PET/CT demonstrated marked focal uptake in GHPA with negligible uptake in a normal brainstem or thalamus, whereas ^18^F‐FDG showed high uptake in both tumors and brainstem tissues (Table 1). The maximal standardized uptake value (SUVmax) and mean of SUV (SUVmean) of ^68^Ga‐pentixafor were significantly different between GHPA and brainstem tissues (*p* < 0.005), while ^18^F‐FGD had a similar uptake in both tissues (Figure 6B,D). Therefore, the maximal tumor‐to‐brain ratio (TBRmax) and mean of TBR (TBRmean) of ^68^Ga‐pentixafor were significantly higher than ^18^F‐FDG (*p* < 0.005) (Figure 6C,E), indicating an enhanced sensitivity. In addition, the SUVmax of ^68^Ga‐pentixafor was significantly correlated with GH levels (*p* = 0.046) (Figure 6F). A trend of correlation between the TBRmean and GH level was also observed (*p* = 0.061) (Figure 6I). There was no correlation between GH and either TBRmax (Figure 6G) or SUVmean (Figure 6H). As for ^18^F‐FDG, neither SUV nor TBR values were significantly correlated with GH or IGF‐1 levels (Figure S9).

**TABLE 1:** Summary of patients’ demographics and clinical characteristics.

Category	Mean (SD)
Age, years	39.86 (9.19)
Gender	Male 2, female 5
Serum GH, ng/mL	17.10 (12.48)
Serum IGF‐1, ng/mL	502.86 (125.70)
Maximal diameter of GHPA, mm	24.00(9.30)
Ki‐67, %	2.57 (1.27)
^68^Ga‐pentixafor pituitary uptake	
Visual positive rate	100% (7/7)
SUVmax^a^	1.53 (0.48)
SUVmean^a^	1.20 (0.57)
TBRmean^a^	12.54 (7.13)
^18^F‐FDG Pituitary uptake	
Visual positive rate	100% (7/7)
SUVmax	6.84 (1.69)
SUVmean^a^	5.05 (1.70)
TBRmean^a^	0.82 (0.27)

^a^ The SUVmax and the TBRmean were the semiquantitative parameters of pituitary adenoma with higher SUVmean in normal brain tissue.

## 3 Discussion

In this study, we established and characterized hPASCs isolated from clinical GHPA patients. The biological characteristics of hPASCs, including their morphology, marker protein expression, and differentiation capacity, were similar to those described in previous reports [7, 10, 11]. Provided that the bulk tumor was primarily composed of terminally differentiated cells [9], we directly compared the genetic profiles of hPASCs and their matched differentiated tumor cells. This comparison can be used to generate a PPI network to reveal potential genetic alterations between the two populations.

MCODE is an efficient method for screening putative related gene modules within PPI networks [25]. This method has been employed to find critical genes in liver cancer [26], lymphoma [27], and glioma [28]. *CXCR4* was ranked as one of the top candidate genes in our analysis. Previous studies have identified CXCR4 expression in pituitary adenomas at both the mRNA and protein levels [18, 19]. Yoshida reported a higher expression of CXCR4 in GHPA than in prolactinoma, nonfunctioning, ACTH‐secreting, and TSH‐secreting pituitary adenomas [17]. Wurth also showed that *CXCR4* was expressed in both hPASCs and differentiated tumor cells [10]; however, no further exploration was performed.

Currently, in the absence of a reliable human‐derived GHPA cell line, rat GH3 cells can represent differentiated GHPA tumor cells and are widely used in vitro GHPA cell model [29]. Furthermore, GH3 cell proliferation was suppressed using the classical CXCR4 inhibitor AMD3100 [17]. Higher suppression efficiency was obtained by using another small‐molecule inhibitor, AMD3465, which is approximately 10 times more potent than AMD3100 [23]. Similar to AMD3100, it reduced the proliferative, invasive, and migratory capacities of GH3 cells. More importantly, AMD3465 significantly impaired hPASC viability, including proliferation, self‐renewal, and differentiation. These findings are consistent with previous reports demonstrating that *CXCR4* suppression inhibits the tumor‐promoting properties of TSCs in other cancers [30, 31, 32]. For example, treatment of meningioma stem‐like cells with a CXCR4 antagonist alone abolished the cellular proliferation, migration, and tube formation induced by CXCL12 [33]. This consolidated the role of *CXCR4* in maintaining the stemness and function of TSCs and potentiating tumorigenesis. Our GSEA analysis further indicated that the PI3K/Akt signaling pathway may be involved in these BPs. This pathway has been verified to regulate the proliferation, migration, and invasion of various cancer types—including ovarian [34], prostate [35], and breast cancers [36]. Consistent with these observations, we showed that the CXCR4‐mediated cellular activities of hPASCs were also regulated by activation of the PI3K/Akt signaling pathway. These findings may provide additional evidence supporting the involvement of the PI3K/Akt signaling pathway in the formation of pituitary adenoma [37].

EMT has been extensively documented as a key driver of tumor invasion and metastasis [38]. Loss of E‐cadherin is a hallmark of this process and is directly associated with poor prognosis and higher tumor grade in multiple tumor types, including pituitary adenomas [39, 40]. We showed that suppressing CXCR4 by AMD3465 significantly reduced the expression of some EMT markers in hPASCs, similar to prior reports describing CXCR4‐mediated EMT in colorectal cancers [41]. We also investigated another aspect of tumor microenvironment, namely angiogenesis, using a GH3 xenograft mouse model. Although the extent of angiogenesis in pituitary adenomas remains debated, increased microvessel density has been observed in macroadenomas and may contribute to invasive behavior [42]. We found that AMD3465 reduced intratumoral microvessel density, consistent with results in other disease models [43, 44]. Collectively, our findings support a pro‐tumoral role for *CXCR4* in remodeling the tumor microenvironment, potentially through a CXCR4‐mediated regulatory network.

In light of this, we continued to investigate the putative *CXCR4*‐associated genes in cluster 1. Clinical GHPA samples verified that *CXCR4* was significantly correlated with mRNA levels of *CXCL12, ICAM1, PECAM1*, and *SELPLG*. Silencing *CXCR4* in GH3 cells directly affected their expression. CXCL12 is the principal ligand for CXCR4, and the CXCL12/CXCR4 signaling axis plays a well‐established role in tumorigenesis [45, 46]. Upon *CXCR4* activation, *ICAM1* can promote adhesion and migration of immune cells and contributes to the progression of several cancers [47]. In pituitary adenomas, ICAM1 was shown to be a downstream effector in the S100A9‐mediated promotion of tumor invasion [48]. *PECAM1* (CD31) is an adhesion molecule expressed on the surface of endothelial cells and platelets that contributes to angiogenesis [49]. Previous studies have shown that PECAM1 participates, either directly or indirectly, in the MFs mediated by CXCR4, thereby acting as an effector of CXCL12 chemokine signaling in different disease models [50, 51, 52]. Our results suggest that reduction of angiogenesis following CXCR4 inhibition may be mediated, at least in part, through PECAM1. SELPLG, also known as P‐selectin glycoprotein ligand‐1 (*PSGL‐1*), is a glycoprotein expressed on the surface of leukocytes and in a variety of cancer types [53]. It has been indicated to participate in cancer metastasis and proliferation [54, 55]; however, its relationship with *CXCR4* has not been defined in tumors. In GH3 cells, silencing *CXCR4* did not alter SELPLG protein level, suggesting that *SELPLG* may not be affected by *CXCR4*. Given the effects of *CXCR4* on hPASCs and the tumor microenvironment, we speculate that the MFs mediated by *CXCR4* are coordinated, at least in part, through a regulatory network involving these downstream genes.

The CXCR4‐targeting radiotracer ^68^Ga‐Pentixafor has exhibited good imaging characteristics in various cancers, such as lymphoma [56], myeloma [57], and breast cancer [58]. To our best knowledge, the study of ^68^Ga‐Pentixafor in assessing GHPA patient is still lacking. Previous studies have shown that ^68^Ga‐Pentixafor exhibited good focal uptake in ACTH‐secreting pituitary adenomas and facilitated the differential diagnosis of ACTH‐dependent and ACTH‐independent Cushing diseases [21]. Since ACTH‐secreting pituitary adenomas and GHPAs represent different tumor subtypes arising from different transcriptional factor lineages, the applicability of ^68^Ga‐Pentixafor in GHPA was evaluated in this pilot study. The parallel comparison between ^18^F‐FDG and ^68^Ga‐Pentixafor in the same patients unequivocally shows that the former lacks specificity for GHPA due to high physiological cerebral uptake, whereas the latter overcomes this limitation by manifesting a high tumor uptake with minimal background uptake, hence possessing an improved lesion detectability and specificity corroborated by TBR metrics. In addition, a significant correlation between ^68^Ga‐pentixafor uptake and GH levels was established, analogous to the correlation between ^68^Ga‐pentixafor uptake and ACTH/cortisol reported by Ding [21]. These findings indicate that CXCR4‐targeted imaging may be related to the tumor cell growth and the hormone‐secreting activity. Although Yoshida reported that *CXCR4* mRNA expression was higher in GHPA than in ACTH‐secreting pituitary adenomas [17], a direct comparison of SUVmax reveals that the uptake value was higher in ACTH‐secreting adenomas [21] than in GHPA. This partially indicates that the uptake of ^68^Ga‐Pentixafor in functioning pituitary adenomas may not be determined solely by *CXCR4* expression. However, factors such as patient demographics, imaging protocols, reference tissue selection, and quantitative imaging parameters may have influenced the results. Further studies directly comparing *CXCR4* expression, hormone secretion, and ^68^Ga‐Pentixafor uptake across multiple pituitary adenoma subtypes are warranted to test this hypothesis.

Although this does not establish that ^68^Ga‐Pentixafor PET/CT is unequivocally superior to conventional MRI in daily clinical examinations, it may offer complementary advantages in assessing tumor growth and secretory activity in pituitary adenomas. Clinically, this approach could potentially aid in outcome prediction and early detection of recurrence. Furthermore, CXCR4‐targeted radionuclide therapy appears to be another promising strategy, with potential applications extending beyond pituitary adenomas to other CXCR4‐expressing malignancies.

A major limitation of this pilot study is the small size of the patient cohort, which precludes further analyses of the translation of the results. Another is that ^68^Ga‐Pentixafor could not be fully evaluated in microadenomas (< 1 cm) because the patients enrolled in this study predominantly had macroadenomas (> 1 cm). Thus, large‐scale, prospective, multicenter clinical trials are needed to address this concern.

## 4 Conclusion

We isolated and cultured hPASCs from GHPA and identified a CXCR4‐mediated cluster that may play a significant role in tumor initiation and progression. As a hub gene, *CXCR4* was able to affect the self‐renewal and differentiation of hPASCs, proliferation and invasion of differentiated tumor cells, and the tumor microenvironment. Using a CXCR4‐based molecular imaging probe ^68^Ga‐Pentixafor, we demonstrated the potential diagnostic value of *CXCR4* in clinical GHPA patients. Our findings indicate that targeting *CXCR4* may be a promising therapeutic strategy for GHPA and other malignancies harboring TSC populations. Taken together, an integrated “CXCR4‐centered diagnosis and therapy” paradigm can be developed to improve clinical outcomes in the future.

## 5 Materials and Methods

### 5.1 Clinical Samples and Information

Eight GHPA clinical samples were collected during neurosurgical procedures and used for hPASC culture and subsequent RNA sequencing (Figure 1A). Detailed clinical information is provided in Table S2. To validate the putative genes related to *CXCR4* predicted from PPI network, an additional cohort of 18 clinical GHPA samples was analyzed using real‐time PCR (Table S3). All samples were pathologically confirmed as GHPA by the clinical pathologists. The study was approved by the Ethics Committee of Beijing Tiantan Hospital, and written informed consent was obtained from all patients.

### 5.2 Human PASC Culture and Verification

The hPASC culture procedure was the same as in a previous report [11]. Each resected GHPA clinical sample during surgery was divided into two portions to ensure a parallel comparison between samples and reduce genetic variance. One portion was stored in liquid nitrogen for pathological immunostaining and sequencing, and the other was processed for hPASC culturing in the laboratory. After washing in 1X phosphate‐buffered saline (PBS), the tumor cells were centrifuged and resuspended to make a single‐cell suspension. This cell suspension was cultured in stem cell medium [DMEM/F‐12 with 2 mM L‐glutamine, 1% penicillin–streptomycin, 1X B27 (50X, Life Technologies, USA), 20 ng/mL basic fibroblast growth factor (bFGF, Peprotech), and 20 ng/mL epidermal growth factor (EGF, Peprotech)] in an incubator at 37°C with 5% CO_2_. The stem cell medium was refreshed every 3 days.

For differentiation, the stem cell medium used for culturing hPASCs was gently removed and the cell spheres were washed with 1X PBS to remove residual medium, and subsequently incubated in pre‐warmed differentiation medium (DMEM/F‐12 supplemented with 15% FBS, 2 mM L‐glutamine, and 1% penicillin–streptomycin) at 37°C in a humidified atmosphere containing 5% CO_2_. Morphological changes were recorded, and cell numbers were counted at designated time points during the differentiation process.

### 5.3 Immunohistochemical Analysis

Cultured hPASCs were seeded on poly‐D‐lysine‐coated plates, washed three times with 1X PBS, fixed in 4% paraformaldehyde for 20 min at room temperature, and permeabilized with 0.3% Triton X‐100 (Sigma‐Aldrich) for 10 min. After three PBS washes, nonspecific binding was blocked with 5% bovine serum albumin for 1 h at room temperature. Cells were incubated with primary antibodies overnight at 4°C, washed, and incubated with Alexa Fluor 488‐ or 647‐conjugated secondary antibodies (Abcam; 1:1000) the next day for 1 h at room temperature.

For tissue immunofluorescence, samples were cryosectioned at 6–10 µm in depth and processed with the same fixation and permeabilization steps. Sections were blocked with 5% serum in 1X PBS containing 0.3% Triton X‐100 and incubated with primary antibodies overnight at 4°C. Next day, the sections were incubated with secondary antibodies for 1 h at room temperature. Nuclei were counterstained with 4',6‐diamidino‐2‐phenylindole (DAPI) for 10 min at room temperature. Fluorescence images were acquired on a Zeiss fluorescence microscope.

### 5.4 Enzyme‐Linked Immunosorbent Assay (ELISA)

ELISA was performed to quantify the level of GH in the culture medium or mouse blood according to manufacturer's instructions (Elabscience, USA). The protocol was consistent with a previous report [11]. The final mixture was evaluated quantitatively at 450 nm using a spectrophotometer (Bio‐Tek, USA).

### 5.5 RNA Extraction and RNA‐Sequencing

Total RNA was extracted using TRIzol reagent following the manufacturer's protocol (Invitrogen, USA). RNA quality and quantity were evaluated with a Bioanalyzer 2100 system (Agilent Technologies, USA), and samples were stored at −80°C until use. RNA sequencing was performed by Novogene Co., Ltd. Double‐stranded DNA was synthesized from cDNA generated from the extracted RNA. Indexed libraries were prepared using the TruSeq PE Cluster Kit v3‐cBot‐HS (Illumina) on a cBot Cluster Generation System and sequenced on an Illumina NovaSeq platform.

Following read alignment, gene expression counts were normalized and analyzed using the “edgeR” package in R software (v3.22.5). *p*‐values were adjusted using the Benjamini–Hochberg method, with *p*_adj_ < 0.05 and |log_2_FC| > 0.5 set as the criteria for identifying DEGs.

### 5.6 Enrichment Analysis

The functional enrichment analysis of DEGs was performed using the clusterProfiler package in R software (v3.22.5) based on the KEGG and GO databases. Three categories are included in GO: BPs, MFs, and cellular components (CCs). A *p*‐value of < 0.05 was considered statistically significant. Enriched KEGG pathways and GO terms were visualized using the ggplot2 package in R software (v3.22.5).

### 5.7 Protein–Protein Interaction and MCODE Analysis

The PPI network of DEGs was predicted using the STRING database (https://cn.string‐db.org) with a confidence score > 0.4. The networks were visualized using Cytoscape (v3.7.2). Significant gene modules were identified using the MCODE plugin with the following parameters: degree cutoff = 2, node score cutoff = 0.2, K‐core = 2, and max depth = 100 [16]. Enrichment analyses of hub genes within each cluster were subsequently performed as described above.

### 5.8 Cell Proliferation Assay

GH3 cells or hPASCs were seeded in 24‐well plates and treated with AMD3465 for the indicated duration. Cell proliferation was assessed using the Cell Counting Kit‐8 (Solarbio, China) according to the manufacturer's protocol. Cell viability was quantified by measuring the absorbance at 450 nm using a spectrophotometer (Bio‐Tek, USA).

### 5.9 Flow Cytometry Assay

GH3 cells were seeded in a six‐well plate at a density of 2 × 10^5^ cells per well in Ham's F12‐K medium in the presence of 2.5% FBS and 15% horse serum (HS), and treated with different concentrations of AMD3465 for 72 h. The cells were washed with 1X PBS, resuspended in staining buffer, and incubated with annexin V‐fluorescein isothiocyanate and propidium iodide (BD Biosciences) for 15 min at room temperature. Samples were analyzed using a CytoFlex flow cytometer (Beckman Coulter), and data were processed using FlowJo software (v10.0, BD Biosciences).

### 5.10 Xenograft Experiment

An in vivo GH3 xenograft model was established using 6‐ to 8‐week‐old female BALB/c nude mice (Charles River, China). The mice were subcutaneously injected with 1.0 × 10^5^ GH3 cells and randomly assigned to three groups 3 weeks post‐inoculation. AMD3465 was administered subcutaneously once daily at doses of 2 mg/kg (low‐dose group) or 4 mg/kg (high‐dose group), with PBS serving as the vehicle. Tumor volume, body weight, and serum GH levels were measured weekly. All mice were euthanized 6 weeks after injection, and the tumors were excised, weighed, and stored at −80°C. Portions of tumor tissue were preserved for immunostaining. All animal procedures were approved by the Institutional Animal Care and Use Committee of the Beijing Neurosurgical Institute.

### 5.11 Sphere Formation Assay

Single‐cell suspensions of hPASCs (1000 cells/well) were seeded in 96‐well ultra‐low‐attachment plates containing stem cell medium. The cells were treated with AMD3465 (50 nmol/L) for 10 days, after which tumor spheres ≥ 50 µm in diameter were manually enumerated.

### 5.12 Western Blots

Total protein (20–50 µg) was extracted from cells or tissues, resolved by 4%–12% sodium dodecyl sulfate‐polyacrylamide gel electrophoresis, transferred to polyvinylidene difluoride membranes, and probed with primary antibodies overnight at 4°C. The next day, the membranes were incubated with horseradish peroxidase (HRP)‐conjugated goat anti‐rabbit or goat anti‐mouse IgG for 1.5 h at room temperature, and signals were visualized by enhanced chemiluminescence using an HRP substrate. The densitometry was performed using ImageJ software (v1.52q).

### 5.13 Transwell Assays

Cell invasion assays were performed using Matrigel‐coated transwell plates (Corning, USA). Briefly, GH3 cells suspended in serum‐free medium were seeded into the upper chamber, while the lower chamber was filled with Ham's F12‐K medium supplemented with 2.5% FBS + 15% HS. After 24 h, cells that migrated to the lower surface of the membrane were fixed using 4% paraformaldehyde and stained with crystal violet. Stained images were acquired using an inverted microscope and cell numbers were quantified using ImageJ software (v1.52q).

### 5.14 Scratch Assay

GH3 cells were seeded into six‐well plates and cultured until reaching 80%–90% confluence. A sterile pipette tip was then used to create uniform vertical scratches across the cell monolayer, guided by a ruler. Following three washes with 1X PBS, the cells were maintained in fresh culture medium. Images were captured at predetermined time points to monitor wound closure.

### 5.15 Knockdown of *Cxcr4* in GH3 Cells

siRNAs targeting *Cxcr4* in GH3 cells and a non‐targeting control (siCtrl) were synthesized by DIA‐UP Biotech (Beijing, China). Cells were transfected with Lipofectamine 3000 (Invitrogen) according to the manufacturer's protocol.

### 5.16 Real‐Time Polymerase Chain Reaction

Total RNA (2 µg) was reverse‐transcribed using the RevertAid First Strand cDNA Synthesis Kit (Thermo Fisher Scientific, USA). Quantitative real‐time PCR (qPCR) was performed with SYBR Green PCR Master Mix (TaKaRa, Japan) on a 7500 Real‐Time PCR System (TaKaRa). GAPDH served as the internal control, and relative expression was calculated using the 2^−ΔΔCt^ method. Primer sequences are listed in Table S7.

### 5.17 Molecular Imaging

Seven preoperative patients with GHPA were enrolled (clinical demographics in Table S4). Each patient had received ^68^Ga‐pentixafor and ^18^F‐FDG PET/CT on separate days before surgery. After surgery, the clinical pathologists verified the pathological diagnosis of GHPA in all patients. Semiquantitative analysis was performed by delineating volumes of interest (VOIs), then extracting SUVmax and SUVmean. TBRmax was calculated as tumor SUVmax divided by brainstem SUVmax, while TBRmean was calculated as tumor SUVmean divided by brainstem SUVmean. This pilot study was approved by the Peking Union Medical College ethics committee, and all participants provided written informed consent.

### 5.18 Statistical Analysis

Statistical analyses were performed using GraphPad Prism 9.0 software (GraphPad Software, Inc., USA). Data are expressed as mean ± standard deviation (SD). A *t*‐test was used to compare the statistical differences. Pearson correlation was used to test for correlated gene expression of *CXCR4*. A *p*‐value of < 0.05 was considered to indicate a significant difference.

## Author Contributions

**Linhao Yuan**: investigation, formal analysis, writing – original draft, writing – review and editing. **Hao Yang**: investigation, formal analysis, writing – review and editing. **Baowang Li**: investigation of PET/CT. **Huimin Sui**: investigation of PET/CT, formal analysis. **Peiliang Li**: clinical resources, draft discussion. **Xu Wang**: methodology. **Jiang Li**: investigation, formal analysis. **Guijun Jia**: clinical resources. **Zhaohui Zhu**: methodology of PET/CT, formal analysis and discussion of PET/CT. **Deling Li**: clinical resources, conceptualization, methodology and supervision of PET/CT. **Wang Jia**: conceptualization, clinical resources. **Peng Kang**: conceptualization, methodology, investigation, supervision, writing – original draft, writing – review and editing. All authors have read and approved the final manuscript.

## Funding

The study was supported by the Natural Science Foundation of Beijing Municipality (7212008). DeLing Li is supported by National Natural Science Foundation of China Youth Fund (82222034). Peng Kang is supported by the Special Fund for the Innovation and Transformation Competition of Beijing Tiantan Hospital (3‐1‐3‐13).

## Conflicts of Interest

The authors declare no conflicts of interest.

## Ethics Statement

Tissue sample collection and processing as well as data collection and use were performed in accordance with local ethics regulations and approvals as well as the 1964 Helsinki Declaration and Its Later Amendments. All procedures were performed in accordance with the ethical standards and approved by the Ethics Committee of Beijing Tiantan Hospital (Ky202215501) and Peking Union Medical College (No. ZS‐1435). All animal procedures were approved by the Institutional Animal Care and Use Committee of the Beijing Neurosurgical Institute (Bni202304005) and conducted in accordance with institutional guidelines.

## Supporting information




**Supporting File**: mco270946‐sup‐0001‐SuppMat.pdf.

## Data Availability

The RNA‐seq datasets generated and analyzed during the current study are available in Genome Sequence Archive (https://ngdc.cncb.ac.cn/gsa/) under project PRJCA056750.
