# Supplementary material for: Transcriptional Profiling of Growth Hormone–Secreting Pituitary Adenoma Stem‐Like Cells: Unveiling the Significant Role of CXCR4 in Tumorigenesis and Clinical Applications
Source: MedComm (2020). 2026 Aug 24;7(9):e70946. doi: 10.1002/mco2.70946 (PMC13501116; doi:10.1002/mco2.70946)
Supplement: Supplementary file 1 [file MCO2-7-e70946-s001.pdf]

Transcriptional Profiling of Growth Hormone-secreting Pituitary Adenoma Stem-like Cells: Unveiling the Significant Role of CXCR4 in Tumorigenesis and Clinical Applications

LinHao Yuan[1#], Hao Yang[1#], BaoWang Li[1], Huimin Sui[3], Peiliang Li[4], Xu Wang[1], Jiang Li[1], Guijun Jia[1], Zhaohui Zhu[3], Deling Li[1,2*], Wang Jia[1,2*], Peng Kang[1,2*]

1. Department of Neurosurgery, Beijing Tiantan Hospital, Capital Medical University, Beijing, China
2. China National Clinical Research Center for Neurological diseases, Fengtai, Beijing, China.
3. Department of Nuclear Medicine, State Key Laboratory of Complex Severe and Rare Diseases, Beijing Key Laboratory of Molecular Targeted Diagnosis and Therapy in Nuclear Medicine, Peking Union Medical College Hospital, Chinese Academy of Medical Sciences, Peking Union Medical College, Beijing, China
4. Department of Neurosurgery, Beijing Ditan Hospital, Capital Medical University, Beijing, China

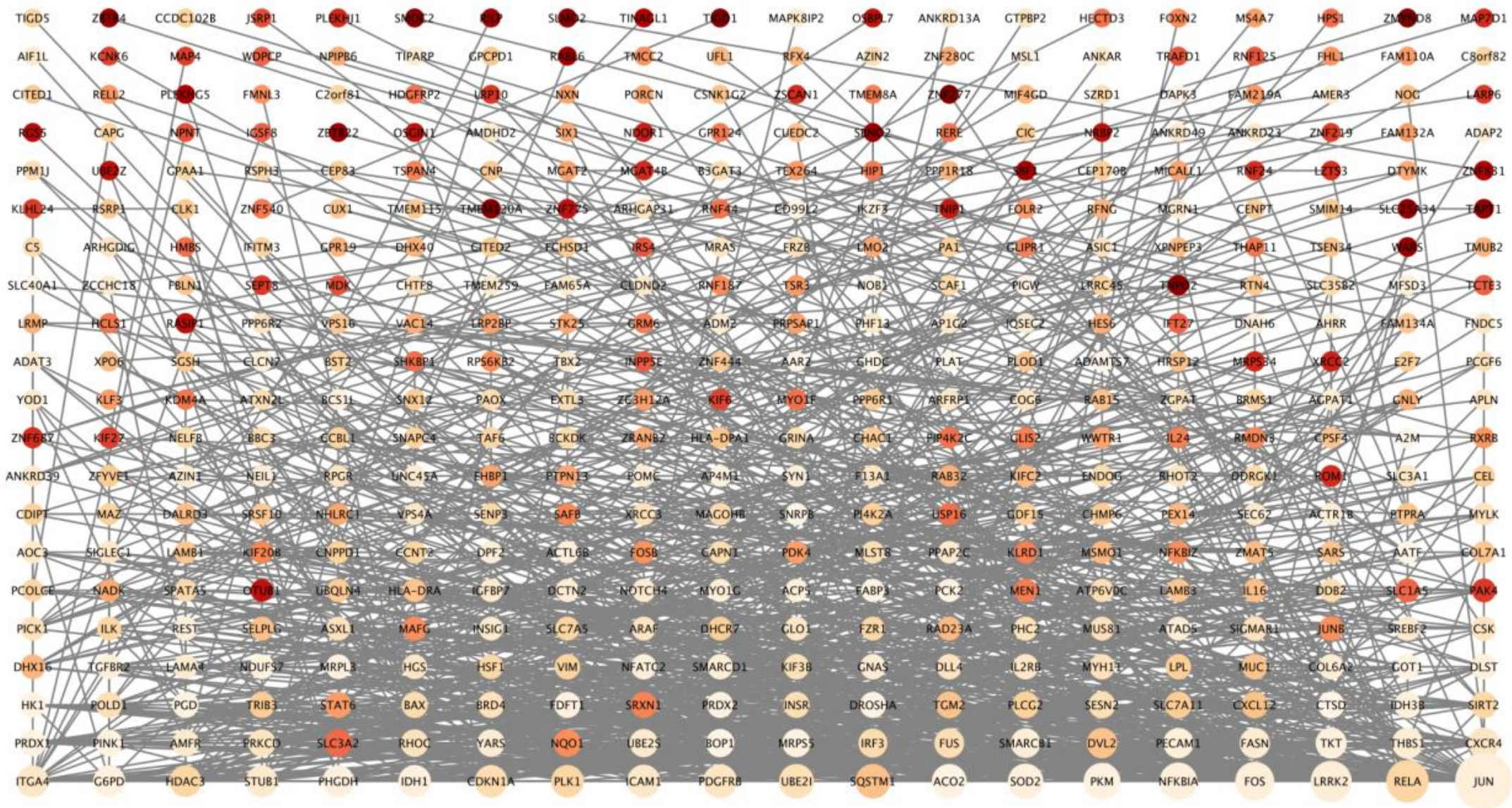

Figure S1. The whole protein-protein interaction (PPI) network of differentially expressed genes between hPASCs and matched bulky tumor samples.

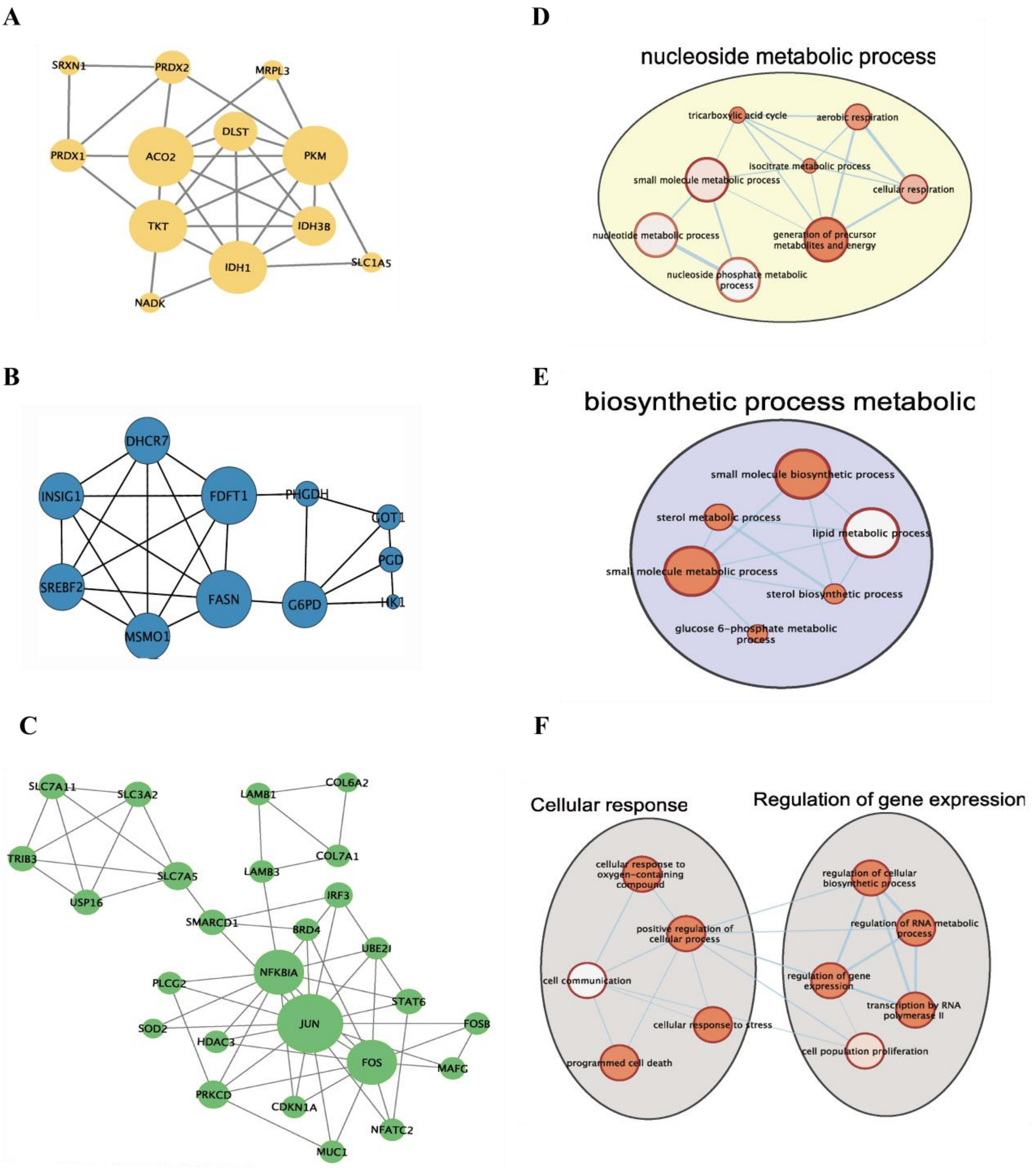

Figure S2. Clusters 2-4 of PPI network predicted by MCODE and their KEGG enrichment analysis. (A) Cluster 2; (B) Cluster 3; (C) Cluster 4; (D) Enrichment of Cluster 2; (E) Enrichment of Cluster 3; (E) Enrichment of Cluster 4.

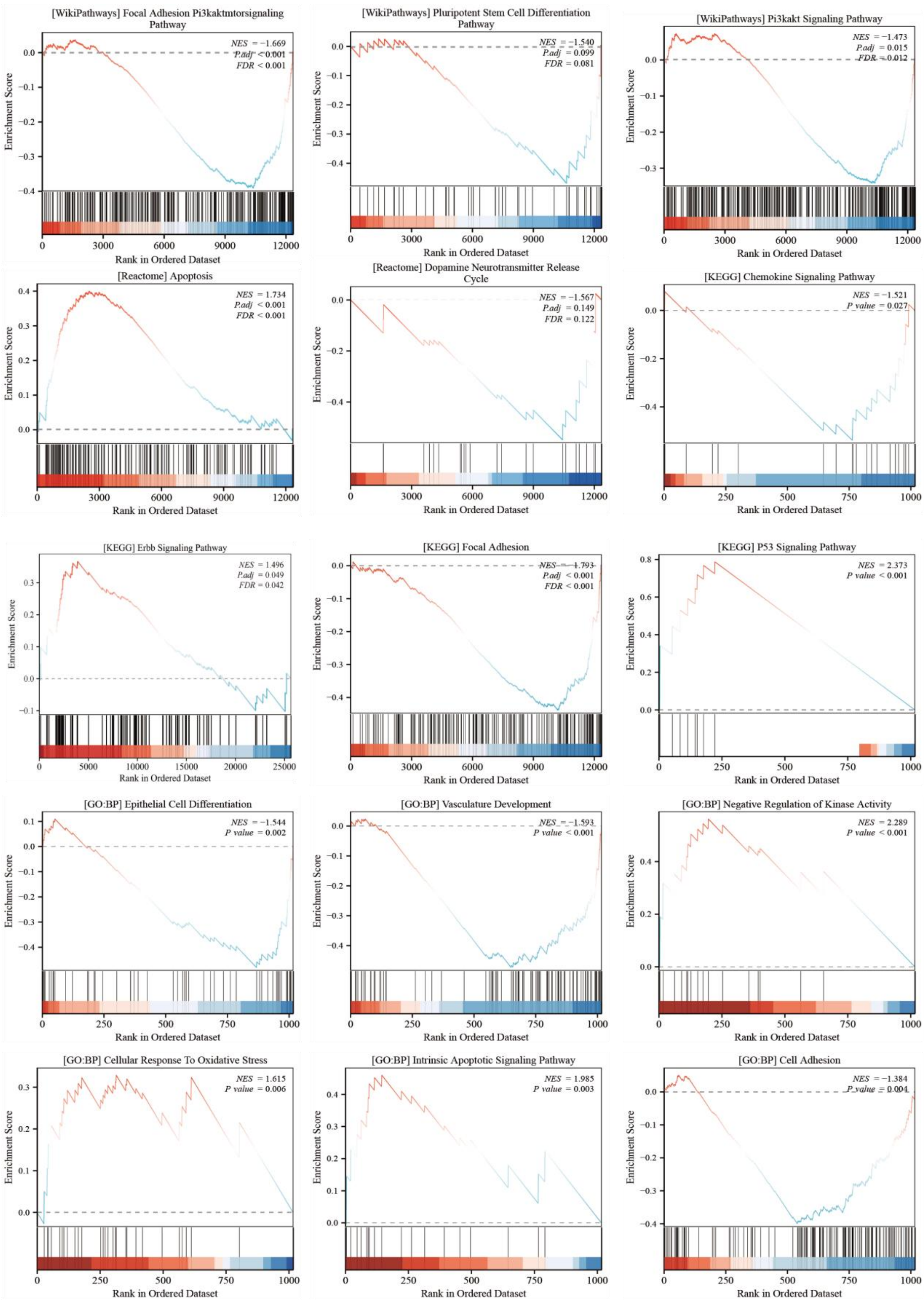

Figure S3. GSEA analysis of differentially expressed genes between hPASCs and bulky tumor cells. Main enrichments of GO, KEGG and signaling pathways are listed.

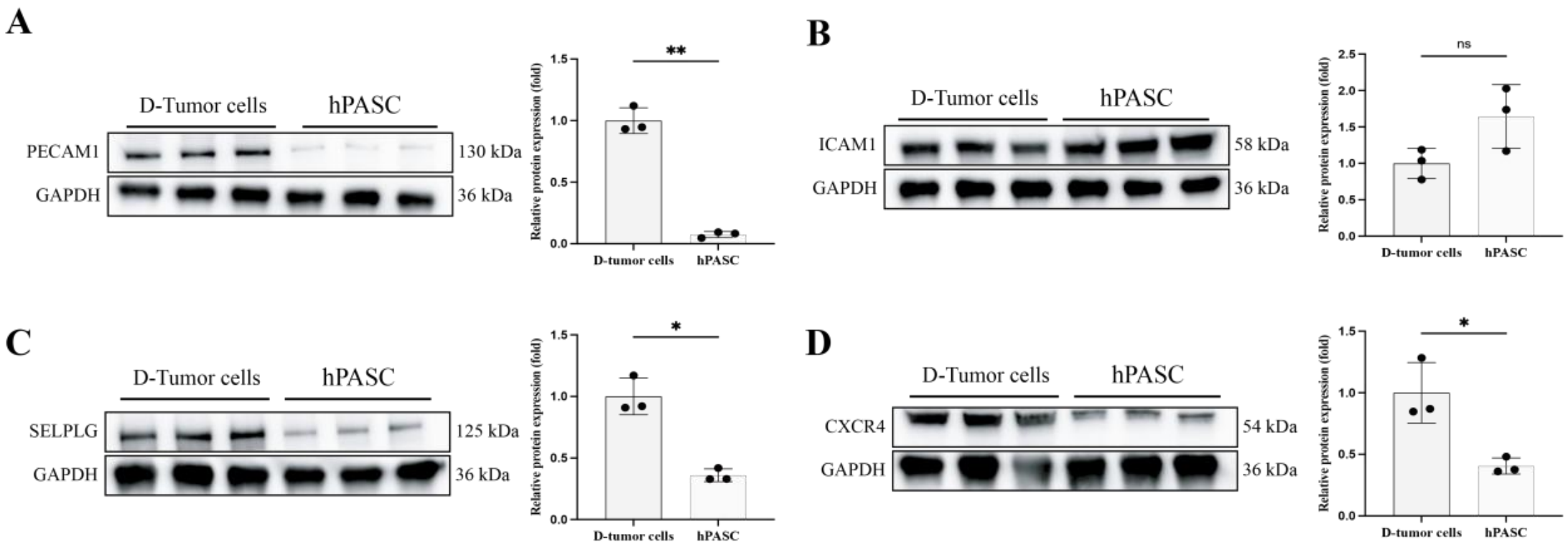

Figure S4. Comparison of protein levels of CXCR4, PECAM1, ICAM1 and SELPLG in hPASC and the differentiated bulky tumor cells by Western blot. (A) PECAM1; (B) ICAM1; (C) SELPLG; (D) CXCR4. D-tumor cells: differentiated bulky tumor cells. * $p < 0.05$; ** $p < 0.01$.

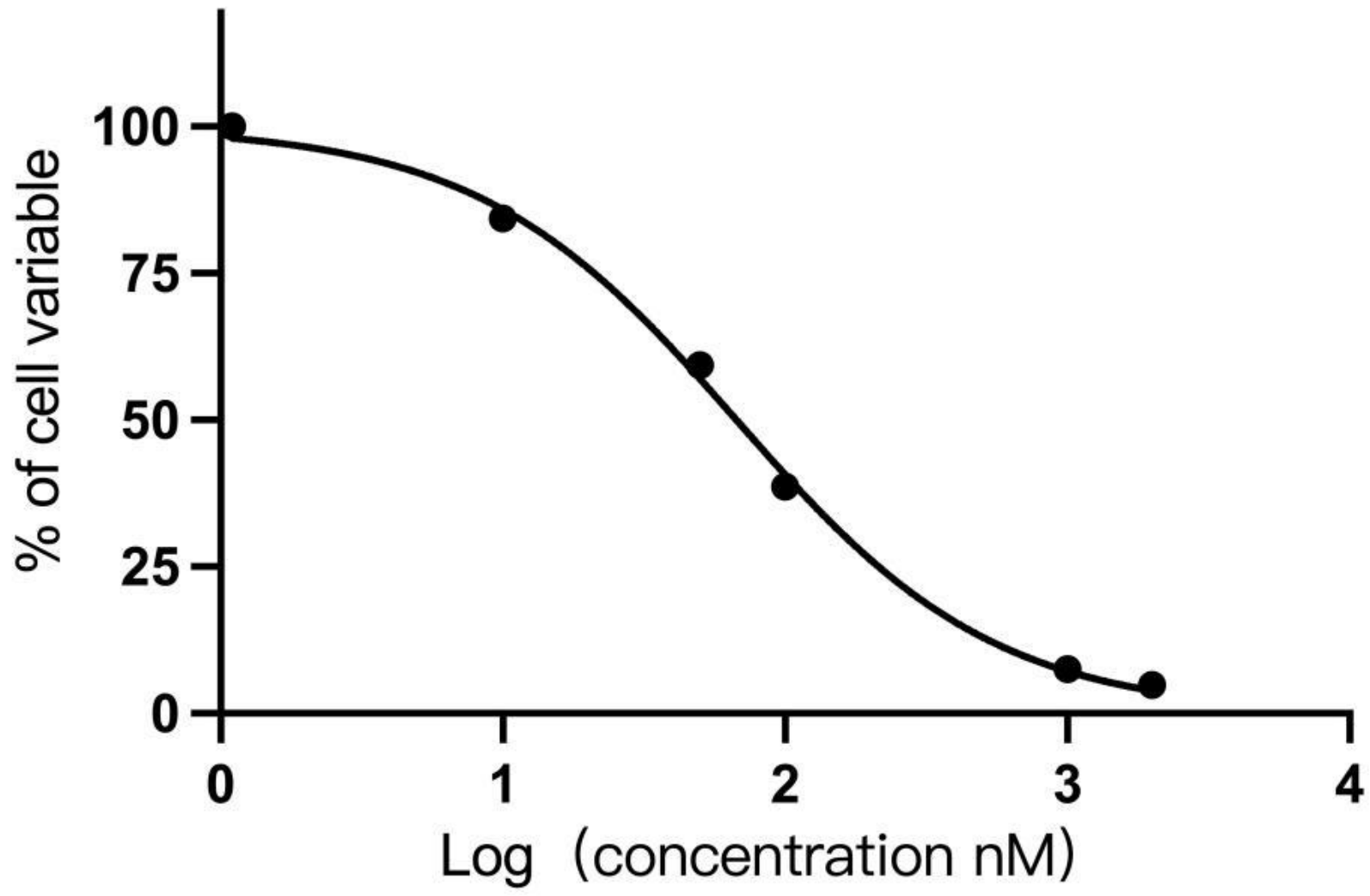

Figure S5. Dose-response curve of AMD3465 in GH3 cells. IC50 is 67nM.

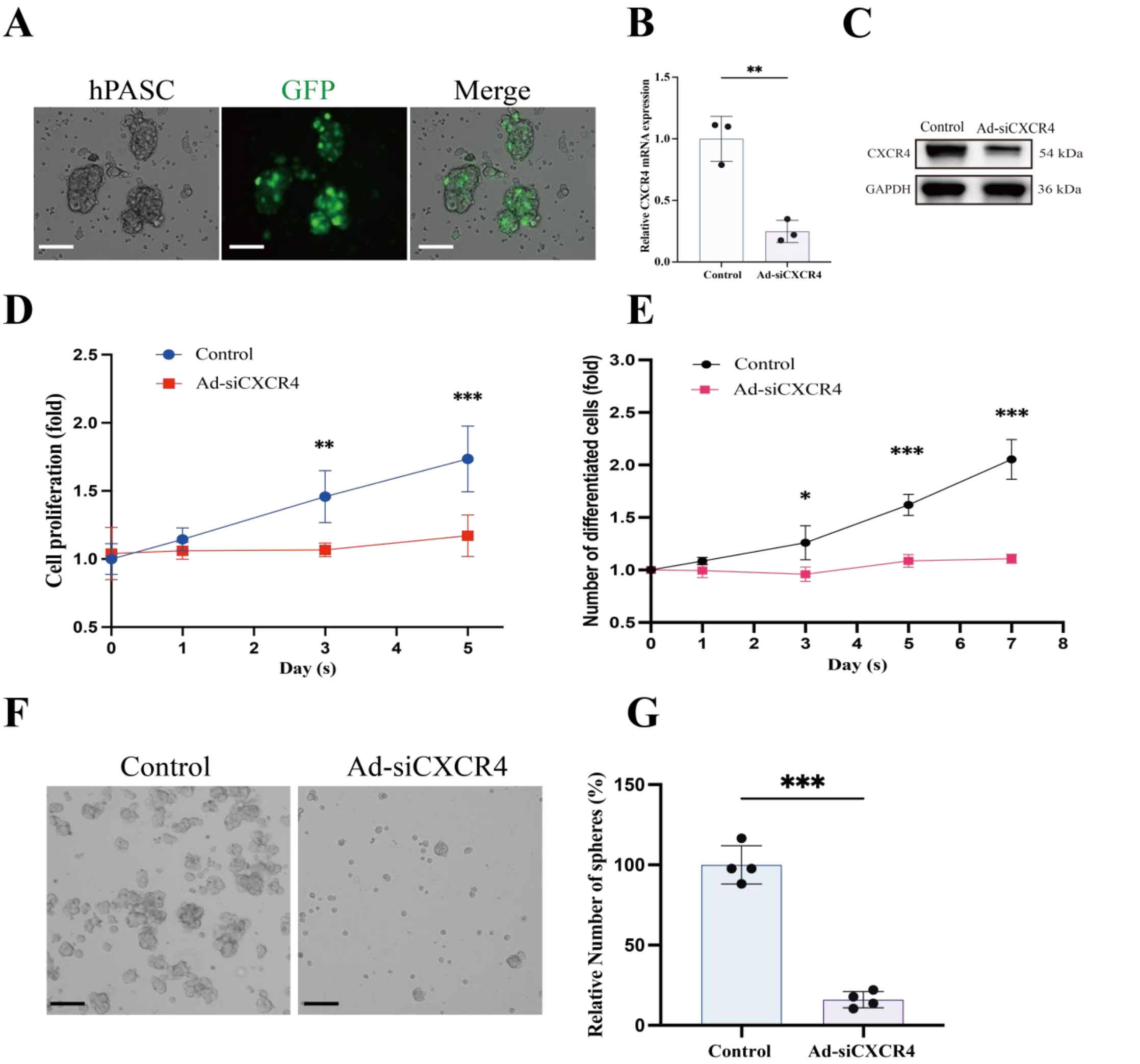

Figure S6. Effects of CXCR4 knockdown on hPASCs. (A) Transfection efficiency of Ad-siCXCR4 vector. (B-C) Silencing efficiency of *CXCR4* in hPASCs measured at (B) mRNA and (C) protein level. N=3 independent experiments. (D) Cell proliferation of hPASCs was significantly reduced in Ad-siCXCR4 group up to 5 days. The reading on Day 0 was normalized as 1. N=3 independent experiments. (E) Number of the differentiated tumor cells from hPASCs was significantly decreased by Ad-siCXCR4 up to 7 days. The reading on Day 0 was normalized as 1. N=4 independent experiments. (F-G) Sphere formation assay showed the number of tumorspheres was significantly reduced in Ad-siCXCR4 group 10 days after transfection. The results of control groups were set as 100%. N=4 independent experiment. Scale bar is 50 um. Ad-siCXCR4: adenoviral-small interference CXCR4. *p<0.05; **p<0.01; ***p<0.001;

A

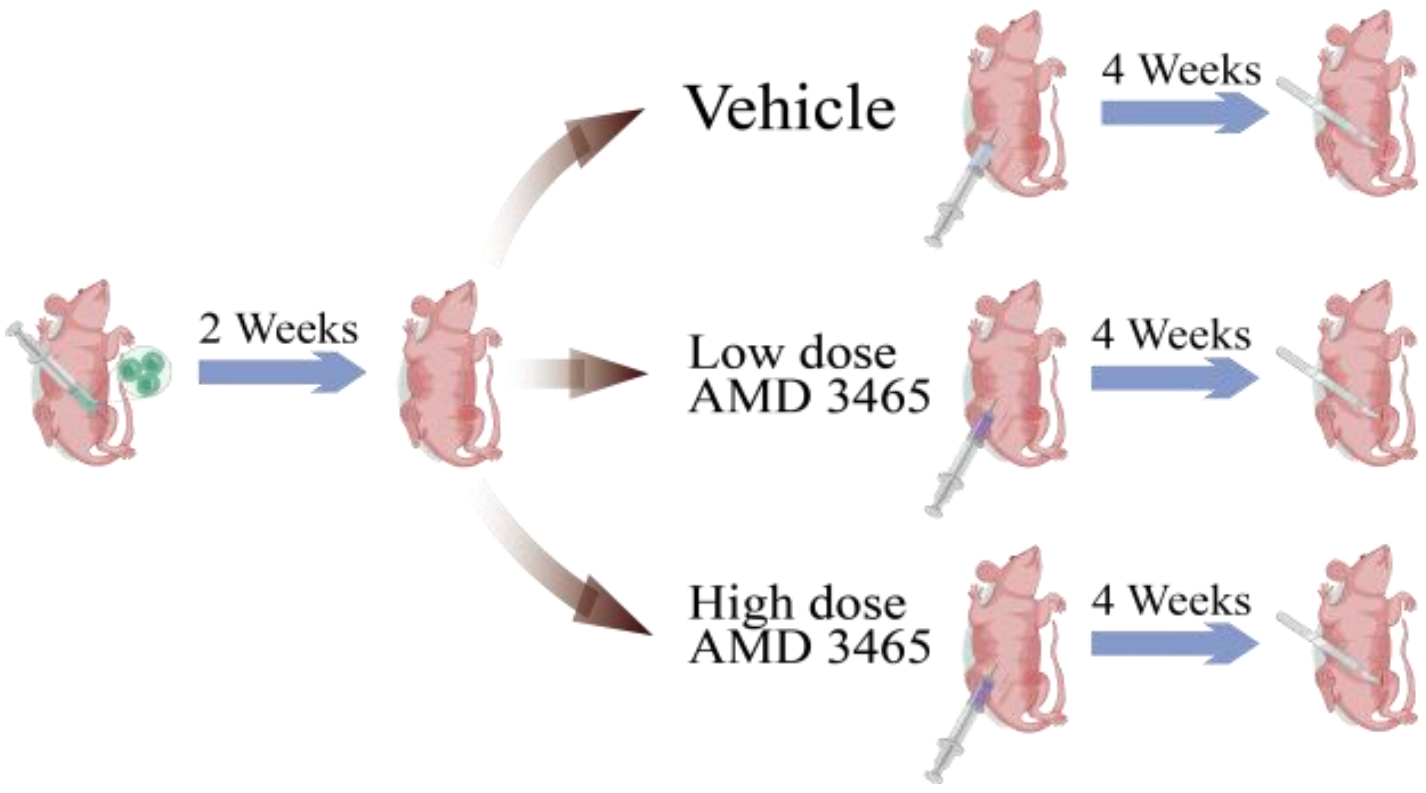

B

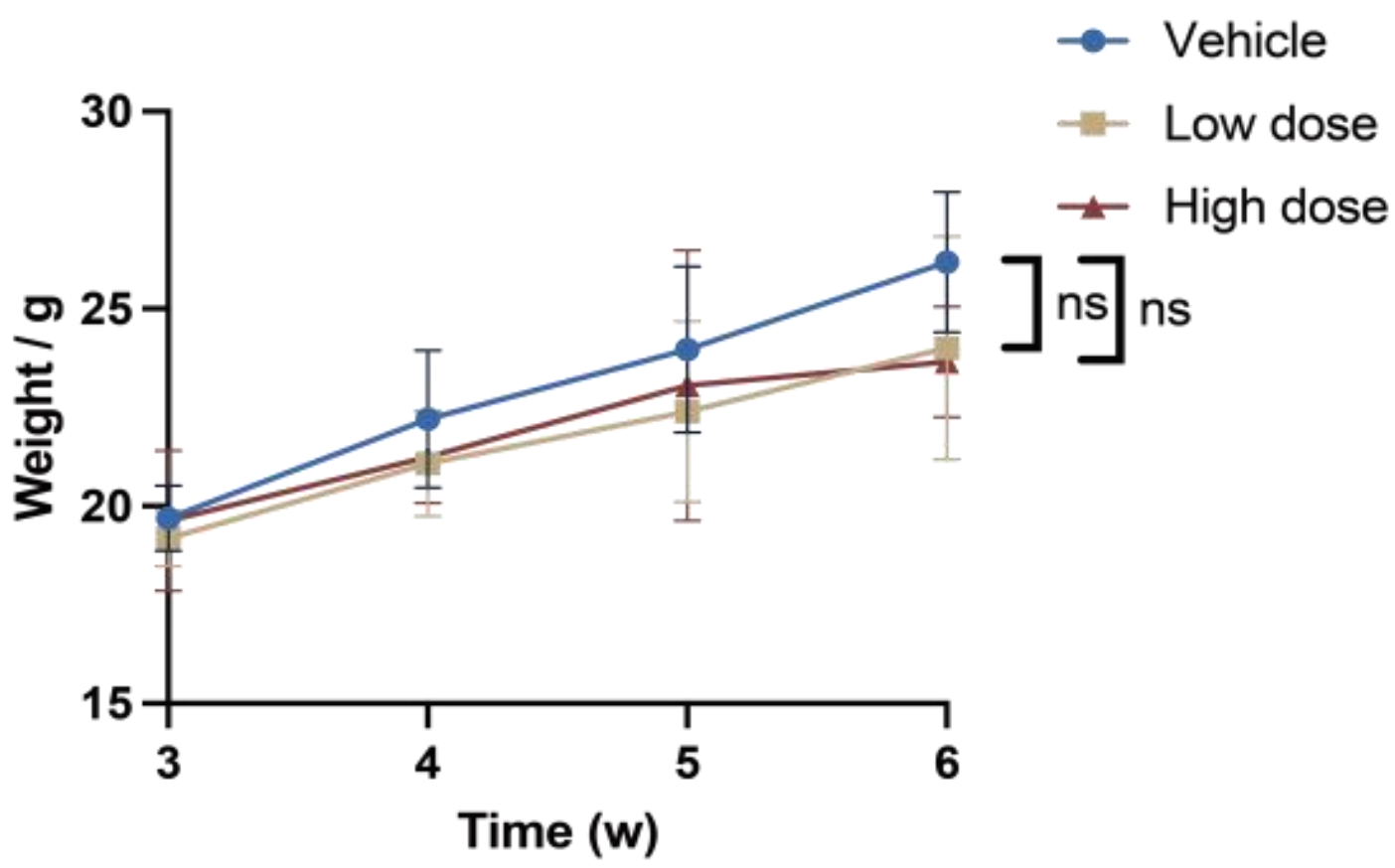

Figure S7. (A) Schematic diagram showing the workflow of experimental procedure of AMD3465 in GH3 xenografted mice. Two concentrations of AMD3465 (low dose 2mg/kg, high dose 4mg/kg) were used. (B) Weight of each GH3 xenograft mouse was measured up to 6 weeks. No significant changes were observed in between vehicle and treatment groups. n=5.

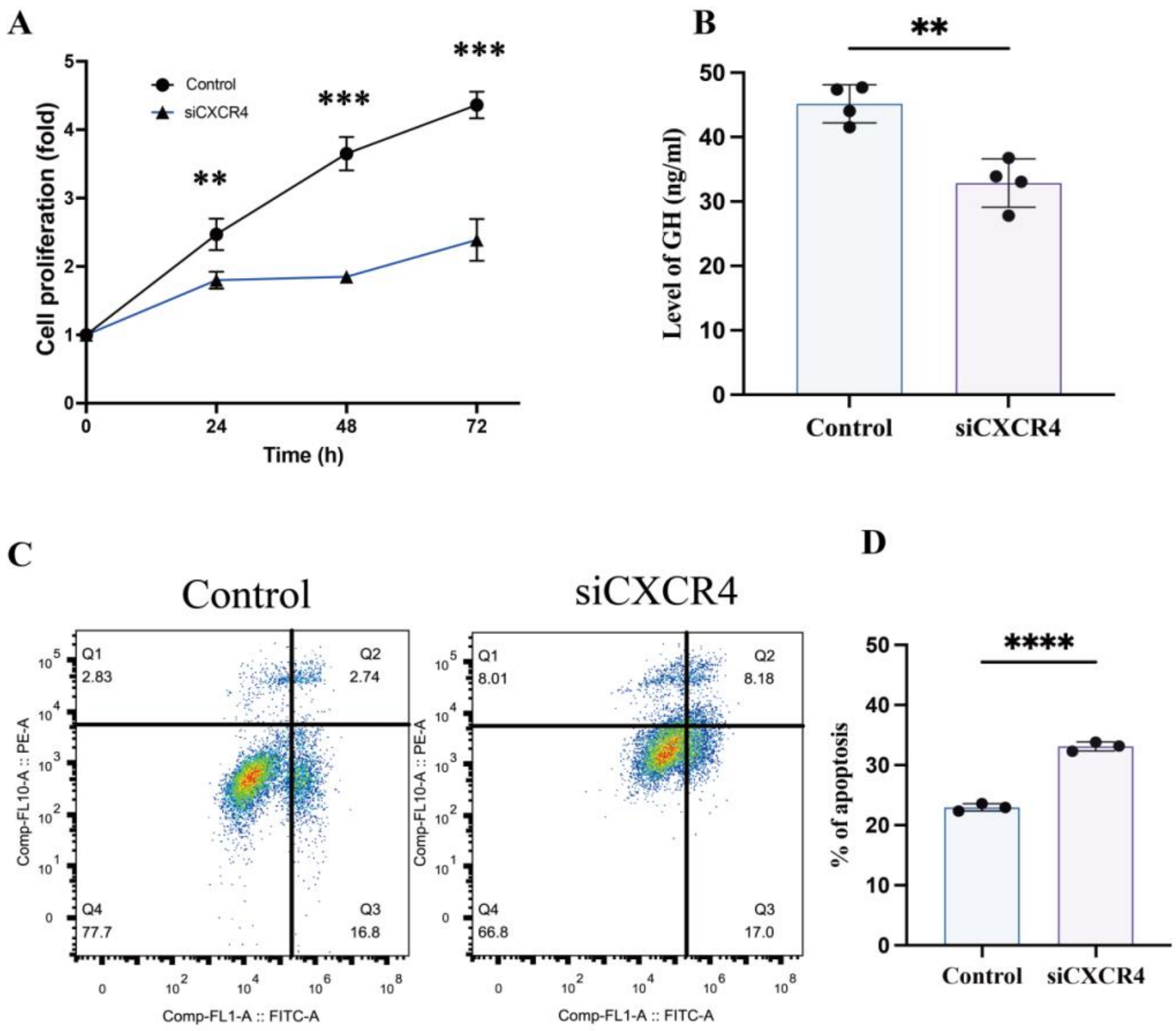

Figure S8. Effects of *Cxcr4* knockdown on GH3 cells. (A) Cell proliferation of GH3 cells was significantly reduced in siCXCR4 group. Day 0 was normalized as 1. N=4 independent experiments. (B) Knockdown of *Cxcr4* in GH3 cells resulted in a significant lower level of GH in the culture medium by ELISA. N=4 independent experiments. (C-D) Suppression of CXCR4 increased the apoptosis rate of GH3 cells quantified by flow cytometry. N=3 independent experiments. $^{**}p < 0.01$; $^{***}p < 0.005$; $^{****}p < 0.001$

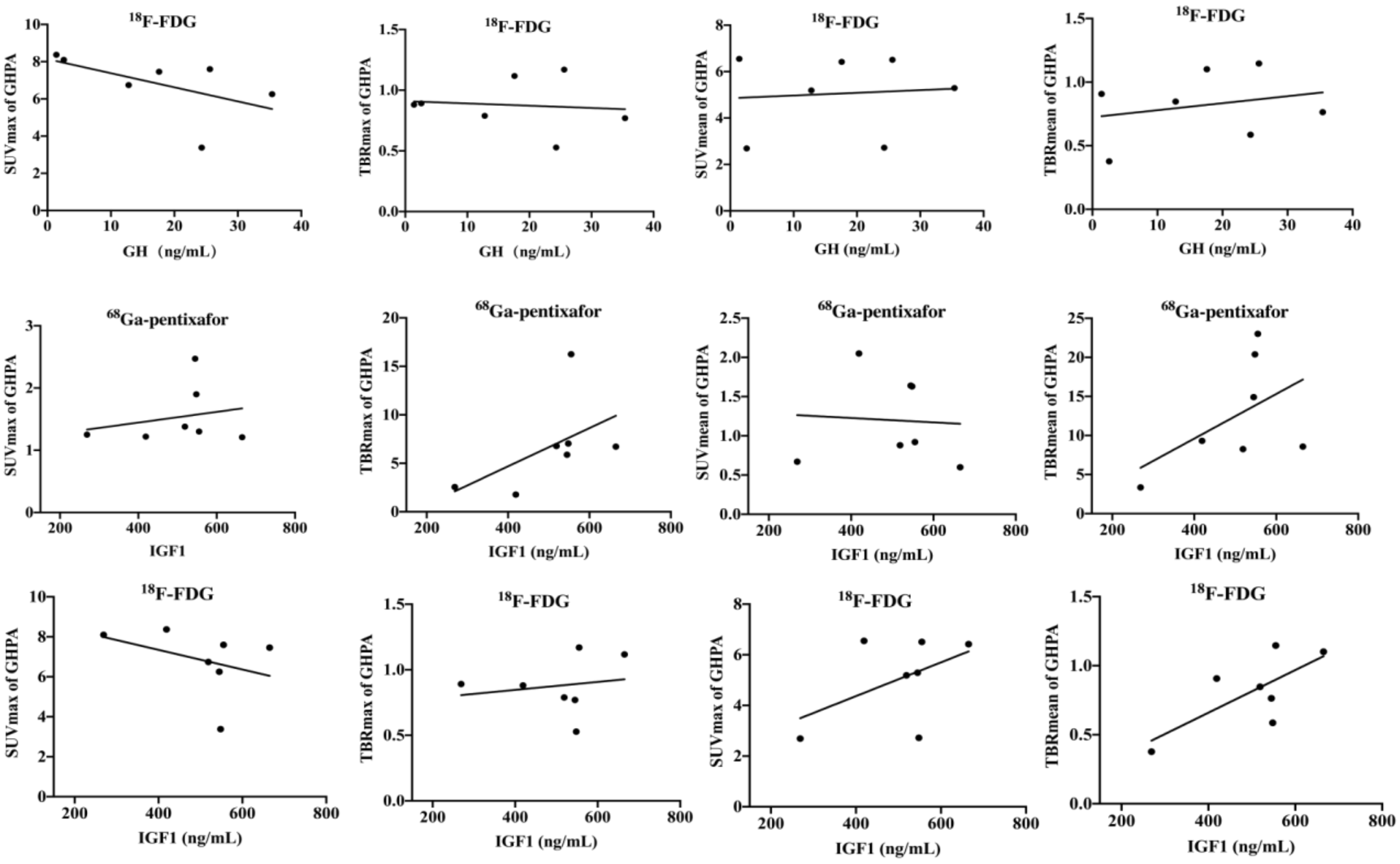

Figure S9. SUVmax, TBRmax, SUVmean and TBRmean of ^{68}Ga-pentixafor and ^{18}F-FDG in correlation with GH or IGF-1 level in human GHPA patients. No significance was observed for both radiotracers.

Table S1. Quantification of putative interacted genes by MCODE

#node1	node2	database_annotated	automated_textmining	combined_score
CXCR4	SELPLG	0	0.501	0.537
CXCR4	THBS1	0	0.408	0.408
CXCR4	PDGFRB	0	0.512	0.512
CXCR4	ICAM1	0	0.665	0.672
CXCR4	PECAM1	0	0.706	0.755
ICAM1	SELPLG	0	0.822	0.829
ICAM1	THBS1	0	0.463	0.491
ICAM1	PDGFRB	0	0.399	0.404
ICAM1	PECAM1	0	0.863	0.87
PDGFRB	THBS1	0	0.374	0.41
PDGFRB	PECAM1	0	0.744	0.75
PECAM1	SELPLG	0	0.455	0.487
PECAM1	THBS1	0	0.601	0.61

Table S2. Clinical demographics of GHPA patients for hPASCs culture and RNA-sequencing

Case	Gender	Age (years)	Pre-operative Recurrence	Maximal diameter of the tumor (mm)	Knosp Grade	GH (ng/ml) *0-8	IGF-1 (ng/ml) #58-219	Pituitary cell lineage (T-Pit, Pit1, SF-1)	Ki67 %	Resection
1	F	35	No	22	4	5	564	Pit1	5	GTR
2	F	51	No	18	4	14.1	699	Pit1	4	GTR
3	F	37	No	31	4	31.6	668	Pit1	2	NGTR
4	F	34	Yes	23	3	0.22	194	Pit1	8	NGTR
5	M	46	No	20	1	32.9	594	Pit1	3	GTR
6	F	37	No	25	2	0.385	247	Pit1	5	GTR
7	M	37	No	22	2	40	836	Pit1	6	GTR
8	M	16	No	26	3	2.88	324	Pit1	6	NGTR

* Normal range of GH

Normal range of IGF-1

GTR： gross total resection

NGTR: non-gross total resection

Table S3. Clinical demographics of GHPA patients for analyzing genes correlated with *CXCR4*

Case	Gender	Age (years)	Pre-operative Recurrence	Maximal diameter of the tumor (mm)	Knosp Grade	GH (ng/ml) *0-8	IGF-1 (ng/ml) #58-219	Pituitary cell lineage (T-Pit, Pit1, SF-1)	Ki67%	Resection extent
1	F	26	No	33	2	32.00	-	Pit1	1	GTR
2	M	29	No	33	4	> 40.00	1543	Pit1	3	NGTR
3	M	59	No	25	3	3.64	629	Pit1	4	GTR
4	F	48	No	16	3	10.8	733	Pit1	4	GTR
5	F	41	No	20	3	19.4	-	Pit1	2	NGTR
6	F	30	No	30	4	41	1149	Pit1	6	NGTR
7	M	34	No	15	1	20.1	-	Pit1	5	GTR
8	F	50	No	10	1	7.83	635	Pit1	2	GTR
9	M	48	No	31	4	41	1220	Pit1	8	NGTR
10	M	55	No	40	2	6.720	-	Pit1	3	NGTR
11	F	37	No	11	1	9.040	-	Pit1	1	GTR
12	M	51	No	21	1	37	-	Pit1	1	GTR
13	F	39	No	22	3	25.6	732	Pit1	6	GTR
14	M	40	No	45	1	41	887	Pit1	3	GTR
15	F	46	No	22	3	23.6	969	Pit1	6	NGTR
16	M	49	No	13	3	15.3	638	Pit1	1	GTR
17	M	56	No	12	3	27.4	365	Pit1	2	GTR
18	F	32	Yes	39	4	9.24	-	Pit1	5	GTR

* Normal range of GH

Normal range of IGF-1

GTR：gross total resection; NGTR: non-gross total resection

Table S4. Clinical information of GHPA patients for preoperative PET/CT examination

Case	Gender	Age (years)	Pre-operative Recurrence	Maximal diameter of the tumor (mm)	Knosp Grade	GH (ng/ml) *0-8	IGF-1 (ng/ml) #58-219	Pathology	Pituitary cell lineage (T-Pit, Pit1, SF-1)	Ki-67 %	Resection
1	F	45	No	20	3	12.8	519	GH	Pit1	2	GTR
2	F	44	No	17	3	35.4	545	GH	Pit1	3	GTR
3	F	36	No	30	3	17.6	665	GH	Pit1	2	GTR
4	F	56	No	42	1	24.3	548	GH	Pit1	1	GTR
5	M	37	Yes	20	2	2.4	419	GH	Pit1	5	NGTR
6	F	32	No	15	2	25.6	555	GH	Pit1	2	GTR
7	M	29	Yes	24	2	2.58	269	GH	Pit1	3	GTR

* Normal range of GH

Normal range of IGF-1

GTR： gross total resection

NGTR: non-gross total resection

Table S5. Listing of siRNA Sequences Targeting CXCR4 Gene in rat and human

Sequence Definition	Sense	Anti-sense
Rat siCXCR4	CCAACAAGGAACCCUGCUU	AAGCAGGGUUCCUUGUUGG
Rat siCtrl	UUCUCCGAACGUGUCACGUTT	ACGUGACACGUUCGGAGAATT
Human shCXCR4	5’-GCCTCAAGATCCTCTCCAActcgagTTGGAGAGGATCTTGAGGC-3’	
Human shCtrl	5’-TTCTCCGAACGTGTCACGTctcgagACGTGACACGTTCGGAGAA-3’	

Table S6. Primer Sequences for Genes Correlated with CXCR4

Gene	Sense Primer	Anti-sense Primer
rat Cxcr4	AGCCTGTGGATGGTGGTGTTC	CCTTGGAGTGTGACAGCTTGGA
rat Pecam1	CAGCGTTCAACAGAGCCAGCAT	GCGGCAATGACCACTCCAATGA
rat Selplg	GCCACCATGTCTGTTCCTCTGT	CCAGGCAGCAGTGTGATGTCA
rat Icam1	CACCTCCTCTGGCTGTCACA	GGTCACGAGTTCACAGTCTTGC
rat cxcl12	TCCGTGGGCTCTGAGTTTTC	CAGGGCCGTCTGTGATCATT
rat Gapdh	TTCACCACCATGGAGAAGGC	GGCATGGACTGTGGTCATGA
Human CXCR4	CCACCATCTACTCCATCAT	ATCTGTCATCTGCCTCAC
Human PECAM1	ATGTGCTGTGAATGAAGGA	ATGTGCTGTGAATGAAGGA
Human SELPLG	ACCAGATAGTGAGAGGATGA	CAGGAGGATGACTTGAAGG
Human ICAM1	CGAGGTGACAGTGAAGTG	CGAGGTGACAGTGAAGTG
Human CXCL12	CCAACGTCAAGCATCTCA	AGGTACTCCTGAATCCACTT
Human PDGFRB	ATGTGACGGAGAGTGTGA	AGGCAGACCAGATGATGT
Human THBS1	GCTCAGTATGACTATGACAGA	CAGGAATGCCATCGTTGT
Human GAPDH	GGCTCTCCAGAACATCATC	ATTCGTTGTCATACCAGGAA

Table S7. List of Antibodies

Antibody	Company	Cat No.
Mouse monoclonal anti-Sox2	Abcam	ab79351
Rabbit monoclonal anti-NESTIN	Abcam	ab105389
Mouse monoclonal anti-CD133	Abcam	Ab19898
Rabbit monoclonal anti-CXCR4	Proteintech	60042-1-Ig
Rabbit monoclonal anti-PI3 Kinase p110 alpha	Cell signaling	#4249
Rabbit polyclonal anti-phospho-PIK3CA (Tyr317)	Affinity	AF4369
Rabbit polycloncal Anti-pan-AKT	Abcam	ab8805
Rabbit monoclonal anti-phospho-AKT1 (Ser473)	MCE	HY-P80276
Rabbit polycloncal anti-CXCL12/SDF-1	Proteintech	17402-1-AP
Rabbit monoclonal anti-E-Cadherin	Cell signaling	#3195
Rabbit monoclonal anti-N-Cadherin	Cell signaling	#13116
Rabbit polyclonal anti-Vimentin	Proteintech	#10366-1-AP
Rabbit monoclonal anti-TWIST1	Cell signaling	#90445
Rabbit polyclonal anti-CD31	Proteintech	11265-1-AP
Rabbit monoclonal anti-VEGFA	Abcam	Ab52917
Rabbit monoclonal anti-ICAM1	Abcam	ab109361
Rabbit monoclonal anti-PECAM-1	Cell signaling	#77699
Rabbit monoclonal anti-PSGL-1	Cell signaling	#47074
Rabbit monoclonal anti-GAPDH	Cell signaling	#2118

Supplemental information

Adenoviral transduction of hPASCs

Recombinant replication-deficient adenoviral vectors carrying short hairpin RNA (shRNA) targeting human *CXCR4* (Ad-siCXCR4) with a green fluorescent protein (GFP) reporter gene was purchased from Shanghai Genechem company. Prior to transduction, hPASCs were seeded into 24-well cell culture plate at an appropriate density and cultured for 6-8 h at 37°C in a humidified atmosphere containing 5% CO_2.

The adenoviral stock was diluted with serum-free medium to achieve a multiplicity of infection (MOI) of 50. An equal volume of diluted adenoviral solution was added to the cells which were incubated at 37 °C with 5% CO for 4-6 h. Then the viral-containing medium was completely removed, and cells were refreshed with stem cell medium under culture condition for 48 h post-transduction. Transfection efficiency was assessed by observing GFP under an inverted fluorescence microscope at 48 h. Then transfected cells were used for quantitation of subsequent experiments, which designated the day as Day 0.
